# Noise-reducing optogenetic negative-feedback gene circuits in human cells

**DOI:** 10.1093/nar/gkz556

**Published:** 2019-07-03

**Authors:** Michael Tyler Guinn, Gábor Balázsi

**Affiliations:** 1 Biomedical Engineering Department, Stony Brook University, Stony Brook, NY 11794, USA; 2 The Louis and Beatrice Laufer Center for Physical and Quantitative Biology, Stony Brook University, Stony Brook, NY 11794, USA; 3 Stony Brook Medical Scientist Training Program, Renaissance School of Medicine at Stony Brook University, Stony Brook, NY 11794, USA

## Abstract

Gene autorepression is widely present in nature and is also employed in synthetic biology, partly to reduce gene expression noise in cells. Optogenetic systems have recently been developed for controlling gene expression levels in mammalian cells, but most have utilized activator-based proteins, neglecting negative feedback except for *in silico* control. Here, we engineer optogenetic gene circuits into mammalian cells to achieve noise-reduction for precise gene expression control by genetic, *in vitro* negative feedback. We build a toolset of these noise-reducing Light-Inducible Tuner (LITer) gene circuits using the TetR repressor fused with a Tet-inhibiting peptide (TIP) or a degradation tag through the light-sensitive LOV2 protein domain. These LITers provide a range of nearly 4-fold gene expression control and up to 5-fold noise reduction from existing optogenetic systems. Moreover, we use the LITer gene circuit architecture to control gene expression of the cancer oncogene KRAS(G12V) and study its downstream effects through phospho-ERK levels and cellular proliferation. Overall, these novel LITer optogenetic platforms should enable precise spatiotemporal perturbations for studying multicellular phenotypes in developmental biology, oncology and other biomedical fields of research.

## INTRODUCTION

Gene expression levels and variability (noise) dictate transcript and protein production that define the properties of living cells in health and disease (1,2). Depending on the interplay between gene function and environmental conditions, expression levels and noise in cellular populations can confer a variety of cellular advantages and disadvantages (3–8). The importance of gene expression noise in cellular processes may be one reason why natural gene regulatory networks contain noise-modulating network motifs (9–11). For example, Negative-Feedback (NF) is a critical and frequent network motif that can reduce noise (11–14) in biological processes as diverse as circadian rhythm, immunological responses or stress signaling in cancer (15–17). Engineering gene circuits that control gene expression levels and noise simultaneously can reveal important thresholds and sensitivities for broad biological phenomena such as metastasis, epithelial-to-mesenchymal (EMT) transition and drug resistance (18).

A few decades ago, bacterial regulator-based systems emerged capable of controlling intermediate gene expression levels (19–21). Despite this advancement, these systems often suffered from high noise since they lacked feedback regulation, therefore leaving gene expression variability as an uncontrolled parameter (22). Adjusting noise has also been neglected by traditional methods of gene expression control, which tend to focus on extreme gene expression changes (e.g. knockout and overexpression) in cells or organisms.

Engineered solutions have emerged more recently using NF in synthetic gene circuits to fine-tune protein expression proportional to an extracellular chemical inducer while also reducing noise (23). However, existing NF gene circuits respond relatively slowly to chemical stimuli and do not enable single-cell level gene expression control (22–25). Optogenetic systems have the potential to overcome these limitations within living cells (26,27). Yet, most optogenetic gene circuits have been studied by transient transfection and their ability to deliver spatiotemporal control with low noise in single cells remains unknown (28,29). Additionally, most existing optogenetic systems are activator-based, incompatible with noise-reduction by *in vitro*, genetic NF (28,29). Currently, NF in optogenetics has only been achieved through *in silico* control of microbes (30,31), leaving genetic, *in vitro* feedback control unexplored, especially in mammalian cells.

Here, as a step toward precisely controlling single-cells with light, we engineer a series of stable human cell lines expressing NF optogenetic gene circuits developed from previously constructed ‘Linearizers’ (23,28). To achieve this, we introduce two novel peptide elements for controlling the Tet-repressor (TetR). We fuse TetR with a light-responsive protein domain (LOV2) (27,32,33), which is further fused to either a Tet-inhibiting peptide (TIP) (34) or a degradation tag consisting of four amino acids (35). These components remain hidden until illuminated, at which point they ensure light-dependent inhibition or degradation of the TetR protein (34–36). The modified TetR regulators repress a stably-integrated fluorescent reporter, enabling light-dependent gene expression measurements. To assess the performance of these novel optogenetic systems, we compare them with the existing optogenetic LightOn system (28) integrated into the same parental cell line. Finally, we also study the versatility of these optogenetic circuits for controlling the mutated KRAS(G12V) gene, relevant to pancreatic and colon cancer (37,38).

Overall, we present novel optogenetic tools that can enable a wide-dynamic range of gene expression in response to light stimuli and ensure low noise for controlling single mammalian cells by using genetic, *in vitro* NF. These synthetic biology tools have the potential to be utilized in single-cell studies of processes as diverse as embryonic development, cancer metastasis and neuron migration.

## MATERIALS AND METHODS

### Transient transfections

Transient transfections had ∼75 000 cells in 500 μl of complete supplemented DMEM media added to each well of a VisiPlate-24 well black plate (PerkinElmer, catalog number: 1450-605) and were first grown for 24 h. After 24 h, 500 ng of total plasmid DNA was added to Opti-MEM™ media (Thermo Fisher Scientific, catalog number: 31985062), P3000 reagent and Lipofectamine 3000 reagent (Thermo Fisher Scientific, catalog number: L3000001), outlined in the Lipofectamine 3000 reagent protocol. Transfection solutions were incubated at room temperature for 10 min and added to the cells where mixing occurred by gently shaking. Analysis of all experiments was performed 24–72 h after transfections were completed.

### Mammalian cell culture

A Flp-In 293 cell line (Thermo Fisher Scientific catalog number R75007) was the parental cell line for all stable-integration cell lines engineered as well as the cell line used for transient transfection. All cell lines were incubated and sustained in a constant environment at 37°C and 5% ${\rm{C}}{{\rm{O}}_2}$. The cell lines were grown in high glucose Dulbecco’s modified Eagle’s medium (DMEM, Thermo Fisher Scientific, catalog number: 11965-092) that was supplemented with 50 ml of 10% fetal bovine serum (FBS, Sigma-Aldrich, catalog number: 12303C), 5 ml of 10 000 units/ml of Penicillin antibiotic and of 10 000 μg/ml of Streptomycin antibiotic (Thermo Fisher Scientific, catalog number: 15140122). Stable cell lines with synthetic gene circuits were maintained under 50 μg/ml of hygromycin drug selection to prevent loss or silencing of genetically integrated payload (Thermo Fisher Scientific, catalog number: 10687010).

### Stable cell-line integration

Gene circuits were introduced into Flp-In 293 cells (Thermo Fisher Scientific catalog number R75007) using lipofectamine 3000 (Thermo Fisher Scientific, catalog number: L3000001) according to company protocol with 3 × 10^5^ cells and 1 μg of plasmid DNA. Cells were transfected with the gene circuit of interest and the Flp recombinase (pOG44, Thermo Fisher Scientific catalog number V600520) plasmid at a ratio of 1:9, respectively. Around 24 h later, cells were washed and given fresh media. Two days later, cells were split to 25% confluency and incubated for several hours, after which hygromycin was added at a concentration of 50 μg/ml. Cells were grown under hygromycin drug selection for several weeks, with media and drug being changed every 3–4 days. After ∼3 weeks, cells were split into T25 filter cap TC flask (USA Scientific, catalog number CC7682-4825) to be frozen down into polyclonal populations or directly sorted into monoclonal populations with flow cytometry. Monoclonal gene circuit populations were expanded and then frozen down according to the Flp-In protocol (Thermo Fisher Scientific).

### Quantitative RT-PCR and quantitative PCR

Seventy-two hours post experimental induction, total RNA was extracted from cells from stable-cell lines created using a RNeasy Plus Mini Kit (Qiagen, catalog number: 74134) following the manufacturer’s protocol. After RNA extraction, reverse transcription was performed using iScript kit (Bio-Rad Laboratories, catalog number: 1708890) following the manufacturer’s protocol. Following RT-PCR, quantitative PCR was performed using TaqMan Fast Advanced Master Mix (Thermo Fisher Scientific, catalog number: 4444557). TaqMan probes for KRAS and GFP were utilized with FAM dye label (Thermo Fisher Scientific, catalog number: 4331182, Hs00932330_m1 KRAS and Mr04097229_mr EGFP/YFP Assay Id, respectively). For normalization, TaqMan probes for glyceraldehyde-3-phosphate dehydrogenase (GAPDH) levels were also utilized with VIC dye label (Thermo Fisher Scientific, catalog number: 4326317E).

### Fluorescence microscopy

Microscopy was performed 24 or 72 h after experimental induction. Cell lines were grown on VisiPlate-24 well black plates and imaged on a Nikon Eclipse Ti-E inverted microscope with a DS-Qi2 camera (14-bit) for phase contrast and fluorescence images. Images were taken with 20× Ph1 objective in phase contrast and GFP mode. The microscope is equipped with Chroma cubes including DAPI 1160B NTE (catalog 49000, Excitation 395/25, Emission 460/50) for DAPI, ET GFP (catalog 49002, Excitation 470/40, Emission 525/50) for FITC/GFP and ET mCH/TR (catalog 49008, Excitation 560/40, Emission 630/75) for TX Red. Experimental data collection and image analysis were performed using Nikon NIS Elements AR v4.40.00 (Build 1084). All images obtained within a single experiment were collected at the same exposure time per field of interest, underwent identical processing and were normalized to the same fluorescent intensities.

### Flow cytometry

Prior to flow cytometry, cells were trypsinized with 0.25% trypsin-EDTA (Thermo Fisher Scientific, catalog number: 25053CI) at 37°C for 5 min. After 5 min, trypsin-EDTA was neutralized with supplemented DMEM, after which cells were filtered and read on a BD Fortessa flow cytometer. About 10 000 cells per sample were collected per experiment.

### Immunofluorescence

Prior to immunofluorescence staining, cells were trypsinized with 0.25% trypsin-EDTA at 37°C for 5 min. After 5 min, trypsin-EDTA was neutralized with supplemented DMEM. Cells were centrifuged for 5 min at 400 *g*. Supernatant was discarded, and cells were resuspended in 1 ml of 4% paraformaldehyde in 1× PBS. Cells were incubated at room temperature for 15 min. Cells were then washed with excess PBS, centrifuged for 5 min at 400 *g*, and supernatant was then discarded. Cells were then incubated in 1 ml of ice-cold methanol for 30 min at –20°C. Cells were then washed with excess PBS, centrifuged for 5 min at 400 *g*, and supernatant was then discarded. Cells were then resuspended in 100 μl primary KRAS (Sigma-Aldrich, catalog number: WH0003845M1) or ERK (Cell Signaling Technology, catalog number: 4370S) antibody at a dilution of 1:800 for 1 h at room temperature in incubation buffer (1× PBS and 0.5 g BSA). Cells were then washed with 1 ml incubation buffer PBS, centrifuged for 5 min at 400 *g*, and supernatant was then discarded. Cells were then resuspended in 100 μl secondary antibody at a dilution of 1:800 for KRAS (Invitrogen, catalog number: A11005) or 1:2000 for ERK (Cell Signaling Technology, catalog number: 8889S) for 30 min at room temperature in incubation buffer. Cells were then washed with 1 ml incubation buffer, centrifuged for 5 min at 400 *g*, and the supernatant was then discarded. Cells were resuspended in 500 μl PBS, filtered and read on a BD Fortessa flow cytometer with 10 000 cells per sample.

### Growth assay

Cells were grown on VisiPlate-24 well black plate at a density of ∼1200 cells per well. Cells were incubated on an LPA for 72 h at different light intensities and sustained in a constant environment at 37°C and 5% ${\rm{C}}{{\rm{O}}_2}$. After 72 h, cells were incubated with NucBlue reagent (Thermo Fisher Scientific, catalog number: R37605) and imaged under the Nikon Eclipse Ti-E inverted fluorescence microscope. Bright spot detection was performed on each replicate under DAPI channel excitation, quantified into number of cellular objects and analyzed for cell number within MATLAB.

### Statistics and reproducibility

Two-sample *t*-tests were performed for significance of fold-change calculations between maximum and minimum expression states of various gene circuits. Additionally, two-sample *t*-tests were performed for analyzing the significance of growth assays, KRAS levels and ERK levels. Kruskal–Wallis test was performed for analyzing significance in CV differences between gene circuits and various light doses.

### Software

Flow cytometry data were analyzed using FCS Express. Fluorescence microscopy data were analyzed using the NIS Elements AR v4.40.00 package. qRT-PCR data were analyzed using Thermo Fisher Scientific’s Relative Quantification App in the Thermo Fisher Cloud. Experimental data were plotted in MATLAB R2018a. Computational models were written in MATLAB & in Dizzy with the parameters given in the Supplementary Information. To test the agreement between the deterministic and stochastic models, we solved for the steady states and performed 10 000 stochastic simulations for each model parameter.

## RESULTS

### Testing the precision of gene expression control with an established optogenetic gene circuit

**Figure 1.**
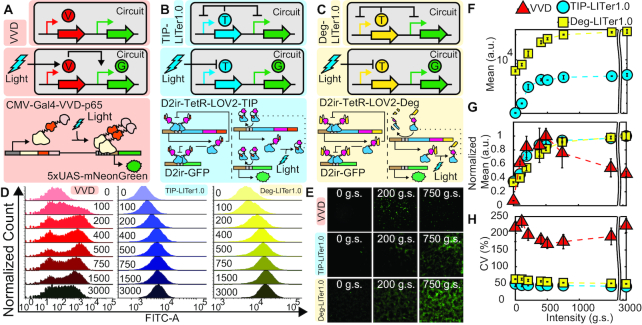
Gene expression control by VVD, TIP-LITer1.0 and Deg-LITer1.0 gene circuits with constant illumination at increasing light intensities. (**A-C**) Simplified and detailed schematics of VVD (**A**), TIP-LITer1.0 (**B**) and Deg-LITer1.0 (**C**) gene circuits stably integrated within the Flp-In 293 cell genome. ‘V’ is the VVD protein, ‘T’ is the TetR regulator with either TIP or Degron and ‘G’ is the Reporter output. (**D**) Fluorescence histograms for flow cytometry performed for each gene circuit under light intensity titration. Numbers within graph represent grayscale value from LPA for stimulating constant light intensity. (**E**) Fluorescence microscopy of cells with corresponding gene circuits over light intensity titration. (**F**) Flow cytometry mean fluorescence versus light intensity titration for the LITer1.0 gene circuits. (**G**) Mean fluorescence values normalized by maximum fluorescence intensity for the three gene circuits. Error bars represent standard deviations; *N* = 3. (**H**) Coefficient of variation (CV) over light intensity titration for the three gene circuits. Two-sample *t*-test was performed on maximum and minimum mean fluorescence data values. TIP-LITer1.0 ON/OFF had a *P*-value of 3.21E-06, Deg-LITer1.0 ON/OFF had a *P*-value of 5.40E-10 and VVD ON/OFF had a *P*-value of 1.28E-04. Kruskal–Wallis test was performed for CV dataset differences. TIP-LITer1.0 versus VVD had a *P*-value of 2.87E-09 and Deg-LITer1.0 versus VVD had a *P*-value of 2.88E-09.

To study the precision of gene expression control for an existing optogenetic benchmark system, we first constructed a stable Flp-In^TM^ 293 cell line integrating the LightOn system expressing the green fluorescence protein, mNeonGreen (Figure 1A) (28). Previous studies have explored gene expression of the LightOn system with transient transfections but have not analyzed stably-integrated mammalian cell lines (39–41). Subsequently, we will refer to this stable engineered cell line as VVD.

To characterize the light responsiveness of VVD cells, we sought a robust illumination platform that could allow a wide-range of tunability for various light parameters. Thus, we turned to the recent light plate apparatus (LPA) system, which is easily customizable for *in vitro* assays, allowing parameter scans for light intensities, pulses and complex light patterns (42). We constructed several LPA systems allowing up to 24 illumination patterns to be probed simultaneously per device (Supplementary Figure S2).

To explore VVD cell response to light, we first analyzed gene expression under increasing light intensities with continuous illumination of the LPA device that has 4096 grayscale (g.s.) levels. We observed nearly 14-fold change of gene expression induction (Figure 1D). However, around the LPA induction of 500 g.s., the response of VVD cells reached a maximum and then dropped for increasing light intensities (Supplementary Figure S3). This behavior has not been described before, potentially because previous studies have utilized custom light apparatuses with limited light probing capabilities. Moreover, we also noticed large gene expression noise, with a coefficient of variation (CV) ∼5-fold higher compared to existing chemically inducible NF gene circuits (23). This observation led us to design light-controllable systems with low noise since high CV may be non-optimal for precise single-cell gene expression control.

### LITer1.0 gene circuits improve the precision of single-cell gene expression control

To achieve lower noise than observed in the VVD cells, we began exploring gene circuit designs that reduce noise. Considering the noise-reducing ability of NF, we moved to integrate negative autoregulation into optogenetic circuits. Notably, most optogenetic systems currently rely on transcriptional activation, which is incompatible with negative autoregulation (28,29). Thus, we turned to existing chemical-inducible mammalian gene expression systems that utilized TetR negative feedback to precisely control GFP reporter expression (23). We hypothesized that upgrading these chemical tools into optogenetic systems could replicate their wide dynamic range of gene expression induction with low noise (23).

To enable NF in optogenetics, we turned toward the blue-light responsive (450 nm) LOV2 protein domain that has been fused to various proteins previously (27,33). We fused the LOV2 protein domain with the repressor protein TetR (Figure 1B) and added the Tet-inhibiting peptide (TIP) (34) capable of inhibiting the TetR protein to the C-terminal end of LOV2. To achieve this, we used the hTetR variant codon optimized for human cells, with a nuclear localization sequence (23). We fused this hTetR with the LOV2 domain by adding a six amino acid sequence (ASGAGA) linker between the two domains. Then, we fused TIP directly with the LOV2 domain in the C-terminal region at the Jα‐helix region without any linker (Supplementary Figure S1). We reasoned that blue light stimulation should open up the LOV2 protein domain, exposing the TIP to bind and inactivate TetR, de-repressing GFP and enabling its expression. Finally, we incorporated this TetR–LOV2–TIP fusion protein under the control of a CMV-based D2ir promoter with two Tet operator (*TetOx2*) sites, allowing feedback regulation (23). Additionally, an identical D2ir promoter drove the expression of the reporter GFP (Figure 1B). We constructed the entire gene circuit into a single vector, stably integrated into Flp-In^TM^ 293 cells and sorted for monoclonal cells. Henceforth, we refer to the cells expressing this gene circuit as TIP-based Light-Inducible Tuner or TIP-LITer1.0.

To complement the TIP-LITer1.0, we next modified the gene circuit by replacing the TIP with a degradation tag consisting of four amino acids (RRRG, Figure 1C) (35). The gene circuit with the degradation tag uses the same hTetR variant, a six amino acid linker (ASGAGA) to fuse the hTetR and LOV2 domains, and the RRRG sequence directly fused with the Jα‐helix region without any linker (Supplementary Figure S1). Like the TIP-LITer1.0 system, the gene circuit is in the OFF-state under darkness. Upon blue light stimulation, the LOV2 protein domain opens and exposes the RRRG tag leading to degradation of the TetR fusion repressor, allowing the expression of GFP. Henceforth, we refer to the cells expressing this gene circuit as Degron-based Light-Inducible Tuner or Deg-LITer1.0.

Next, to compare the characteristics of these new gene circuits with an existing optogenetic circuit, we performed the same light intensity dose–response measurements on TIP-LITer1.0 and Deg-LITer1.0 as we did with the VVD system. We observed a wide dynamic range of gene expression induction (Figure 1D and E). Importantly, at the population level, we observed much tighter fluorescence distributions for both the TIP-LITer1.0 and the Deg-LITer1.0 than for VVD, indicating lower gene expression noise.

For quantitative comparisons of the three gene circuits’ gene expression levels, we examined the mean fluorescence output at full induction (Figure 1F and G). Interestingly, the Deg-LITer1.0 had the highest expression, the TIP-LITer1.0 had an intermediate expression, and the VVD had the lowest level of expression. We achieved ∼3-fold stable gene expression fold-induction for all light doses for both LITer1.0 systems as opposed to the VVD system, which became inactivated at prolonged periods of high-intensity light exposure (Supplementary Figure S3). Consequently, we performed subsequent measurements on the VVD system at 24-h terminal time-points and the LITer1.0 systems at 72-h terminal time points.

To compare the noise of the three systems, we examined their CV. Strikingly, we observed up to 5.5- and 4.5-fold lower CV of TIP-LITer1.0 and Deg-LITer1.0, respectively, compared with the VVD system (Figure 1H). We hypothesize three possible sources for this noise reduction. First, negative feedback is known to reduce gene expression noise (12,43). Second, the slow photocycle kinetics of the VVD protein versus the fast photocycle kinetics of the LOV2 protein domain may lead to higher variation among induced VVD cells (44,45). Third, monomers of the LOV2 protein domain can induce gene expression while VVD activation requires dimerization, possibly increasing cooperativity and noise amplification.

Considering the dose–response linearity of chemically inducible NF gene circuits (23) and to explore the use of LITer1.0 gene circuits for analog gene expression control, we also inspected the linearity of their light intensity dose–response (Supplementary Data). The LITer1.0 dose–responses were approximately linear up to 225 g.s. from the LPA (Supplementary Figure S4), complementing other systems that exhibit linearity with pulsed illumination (46). The linearity was reduced in the LITer1.0 gene circuits compared to chemically inducible systems, because of two main dose–response features. First, the gene circuit did not respond at very low light intensities, causing the dose–response to start off flat (Supplementary Figure S4). Second, even after this flat part, the dose–response did not remain linear for a wide inducer range, starting to curve downwards at 225 g.s. (Supplementary Figure S4). To investigate the sources of these deviations from standard linearizer characteristics, we generalized earlier deterministic models (23), allowing basal gene expression, reversion of induced LOV2 into its inactive state and adjustment of the Hill parameters. Indeed, elevated basal gene expression explained the flat start-off, while Hill parameter adjustments captured the reduced linearity (Supplementary Figure S5 and Supplementary Data). Additionally, increased refolding of LOV2 into an inactive state increased the light needed to induce a specific level of GFP. When combining all these parameters into a single model (Supplementary Figure S5), the dose response more closely matched the experimental results.

Finally, to examine potential clonal effects on gene circuit behavior in the engineered cell lines, we compared dose–responses of polyclonal versus monoclonal populations of the VVD, TIP-LITer1.0 and Deg-LITer1.0 cell lines (Supplementary Figure S6). Since each polyclonal population consists of multiple clones, if clonal isolates affect gene circuit behavior then the polyclonal and monoclonal dose–responses should differ from each other. Remarkably, the monoclonal populations had nearly identical dose–responses to the polyclonal populations, despite these latter populations consisting of many clones. Accordingly, the dose–responses of mean fluorescence intensity and gene expression noise for monoclonal and polyclonal populations were very similar, indicating minimal clonal effects on gene circuit behavior. This is consistent with the minimal clonal effects and variation of the Flp-In 293 cell line observed by Thermo Fisher Scientific and by other research groups (47) as the genomic FRT site location (48) is identical in all cells (e.g. descended from parental Flp-In 293 cells).

In the Flp-In system, a promoter (promoter trap) initially driving the expression of a LacZ-Zeocin fusion protein is part of the genomic integration site. Proper plasmid integration at the genomic FRT site ensures that the hygromycin resistance gene inserts downstream from the promoter trap replacing the LacZ-Zeocin expression, allowing drug selection for cells with proper integration. Cells with random integration die since their hygromycin genes will have low chance for a native promoter to drive hygromycin resistance gene transcription. Since the plasmid backbone design is the same for all gene circuits involved, drug selection enforces that each gene circuit integrates and remains within the same genomic locus for all cells, which we ensure by maintaining constant hygromycin exposure for all cell lines.

Overall, these intensity dose–response experiments provide initial characterization of a classical and two newly engineered optogenetic systems, which we next explored under pulsed light-induction regimes.

### Responses of VVD and LITer1.0 gene circuits to pulsed inputs

**Figure 2.**
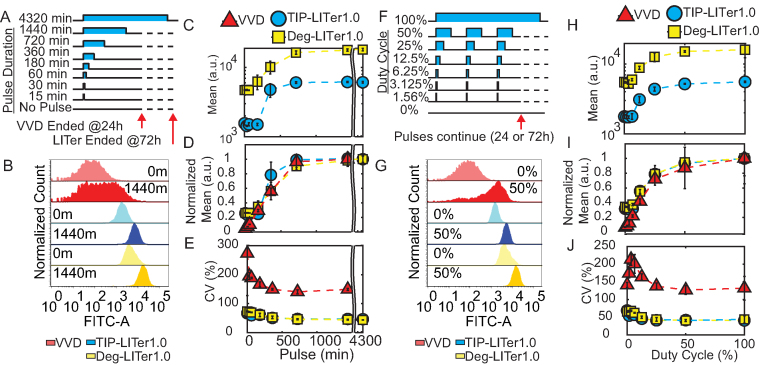
Gene expression control by VVD, TIP-LITer1.0 and Deg-LITer1.0 gene circuits with pulsed illumination. (**A**) Schematic illustration of single pulse experiment. (**B**) Fluorescence histograms for single light pulse titration. Numbers on histograms represent minutes of single pulse duration. (**C**) Flow cytometry mean fluorescence expression versus pulse length for single pulse experiment of LITer1.0 gene circuits. (**D**) Normalized mean fluorescence values versus pulse length for single pulse experiment. (**E**) Coefficient of variation (CV) versus pulse length for single pulse experiment. (**F**) Schematic illustration of duty cycle experiment. (**G**) Fluorescence histograms for duty cycle experiment. Percentages on histograms indicate duty cycles (fraction of each 1 h period for which light was on). (**H**) Flow cytometry mean fluorescence versus duty cycle for LITer1.0 gene circuits. (**I**) Normalized mean fluorescence values versus duty cycle. (**J**) CV versus duty cycle. Error bars represent standard deviation; *N* = 3. Two-sample *t*-test was performed on maximum and minimum mean fluorescence data values for pulse and duty-cycle experiments. TIP-LITer1.0 ON/OFF had a *P*-value of 1.76E-06, Deg-LITer1.0 ON/OFF had a *P*-value of 7.77E-06 and VVD ON/OFF had a *P*-value of 7.03E-05 for single pulse experiment. TIP-LITer1.0 ON/OFF had a *P*-value of 3.17E-06, Deg-LITer1.0 ON/OFF had a *P*-value of 0.0083 and VVD ON/OFF had a *P*-value of 0.0092 for duty-cycle experiment. Kruskal–Wallis test was performed for CV dataset differences. TIP-LITer1.0 versus VVD and Deg-LITer1.0 versus VVD had a *P*-value of 2.88E-09 for the pulse experiment. TIP-LITer1.0 versus VVD had a *P*-value of 2.88E-09 and Deg-LITer1.0 versus VVD had a *P*-value of 2.87E-09 for the duty-cycle experiment.

To characterize the response of VVD and LITer1.0 gene circuits to discontinuous light-induction regimes, we next investigated by fluorescence microscopy and flow cytometry measurements how single light pulses of variable duration and fixed LPA intensity (1000 g.s.) affect gene expression (Figure 2A and Supplementary Figure S7).

We found nearly 4-fold change of gene expression induction for the LITer1.0 gene circuits compared to 21.9-fold for VVD for different pulse lengths. Interestingly, VVD expression saturated for pulsed illumination (Figure 2B–D), implying that shorter (<24 h) illuminations and intermediate intensities eliminate the fall in activity at high doses with continuous illumination. Importantly, we also observed around 4-fold lower gene expression noise for both LITer1.0 gene circuits compared with the VVD system (Figure 2E).

As another discontinuous light-induction regime, we tested the effects of varying the duty cycle (percent of time ON) for periodic light stimuli on the gene circuits. We chose a stimulus period of 1 h and measured gene expression at increasing duty cycle percentages (Figure 2F). Again, we found broad distributions in the VVD system and tight distributions for the LITer1.0 systems (Figure 2G). The VVD system could achieve around 2-fold induction with as little as 6% duty cycle for which the LITer1.0 systems required 12.5% duty cycle (Figure 2H and I). As before, the CVs of the LITer1.0 gene circuits were several-fold lower (Figure 2J).

Interestingly, low duty cycles could achieve near maximal expression implying there may be benefits to low ON/OFF ratios versus continuous light, perhaps due to cellular toxicity at long exposures (49,50). Overall, the LITer1.0 gene circuits had lower noise than VVD, but their basal expression was high for expressing functional genes. To optimize these optogenetic circuit prototypes for functional gene expression, we next turned to computational modeling to reveal experimental parameters that could be adjusted to lower basal expression.

### Computational models suggest improvements for LITer1.0 gene circuits

To examine strategies for optimizing the LITer1.0 architecture by lowering basal expression, we asked if reducing the system from two promoters to one promoter would lower the basal expression. We reasoned that decreasing the number of *TetO* operator sites competing for TetR might increase the effective time for which TetR would be bound, and would lead to stronger repression of GFP expression. Therefore, we developed deterministic and stochastic models of basal expression in double-promoter LITer1.0 systems versus single-promoter LITer2.0 systems, considering a set of simple reactions including transcription, translation, degradation, TetR binding, TetR unbinding and expression leakage (Supplementary Figure S8A and B). The deterministic model using identical parameters for all genes based on previous models (23) (Supplementary Figure S8C, and Supplementary Tables S2 and S3) indicated no difference between LITer1.0 versus LITer2.0 systems except when allowing DNA-bound TetR to degrade. A stochastic computational model using the Gillespie algorithm in MATLAB and the Dizzy software package for validation (Supplementary Data) (51–53) matched the deterministic model, showing insignificant changes in basal expression for the selected parameters (Supplementary Figure S8C).

To more realistically match experimental conditions, we then began exploring changing parameters between GFP and TetR synthesis. Despite both GFP and TetR having the same promoters (D2ir), equal parameters in the LITer1.0 models were not realistic because only GFP contains a Kozak sequence and an intron in the 5′ region to increase translational and transcriptional efficiencies respectively, compared to the TetR protein. We therefore moved to explore how such differential transcriptional and translational efficiencies affected the basal expression of GFP in LITer1.0 versus LITer2.0 systems. Thus, to reflect the lack of a TetR intron, we set TetR transcriptional synthesis and leakage rate at 50% of GFP in LITer1.0, obtaining a clear increase in basal GFP expression versus the LITer2.0 system (Supplementary Figure S8D). Next, to reflect the lack of a Kozak sequence, we set TetR translational synthesis rates at 50% of GFP in LITer1.0 (Supplementary Figure S8E), which increased basal GFP expression further versus the LITer2.0 systems. These results indicate two mechanisms that should diminish high basal expression upon converting LITer1.0 into LITer2.0 systems.

Finally, we applied both parameter modifications simultaneously to test if greater basal expression reduction in LITer2.0 could occur (Supplementary Figure S8F). Indeed, the basal expression decreased more than for each individual parameter change alone. Overall, these models predicted that co-transcribing TetR and GFP would equalize the transcription and translation rates of TetR and GFP, suggesting simple molecular cloning modifications to lower the basal expression of a GOI. Next, we tested these predictions experimentally.

### LITer2.0 gene circuits improve controllability and applicability

**Figure 3.**
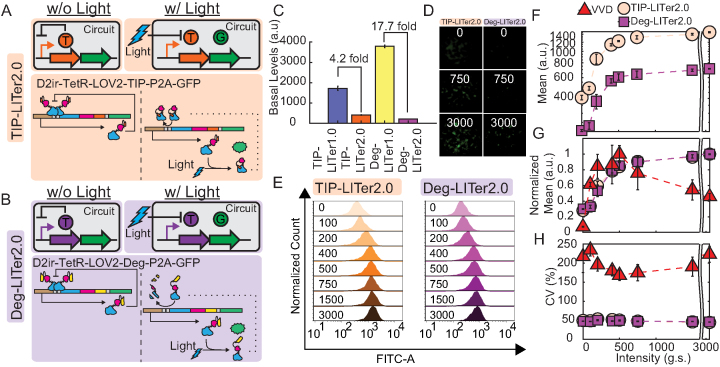
Gene expression control by VVD, TIP-LITer2.0 and Deg-LITer2.0 gene circuits with constant illumination at increasing light intensities. (**A**,**B**) Simplified and detailed schematics of the TIP-LITer2.0 (**A**) & Deg-LITer2.0 (**B**) gene circuits, where w/o is without light and w/ is with light. LITer2.0 gene circuits were stably integrated within Flp-In 293 cell genome. ‘T’ is the TetR regulator with either TIP or Degron and ‘G’ is the Reporter output. (**C**) Basal expression of LITer1.0 gene circuits vs LITer2.0 gene circuits. (**D**) Fluorescence microscopy of cells with corresponding gene circuits over light intensity titration for the LITer2.0 gene circuits. (**E**) Fluorescence histograms from flow cytometry performed on cells with corresponding gene circuits under light intensity titration. Numbers within graph represent grayscale values from the LPA for induction with constant light intensity. (**F**) Flow cytometry mean fluorescence versus light intensity titration for the LITer2.0 gene circuits. (**G**) Mean fluorescence values normalized by maximum fluorescence level versus light intensity for the three gene circuits. (**H**) Coefficient of variation (CV) versus light intensity titration for the three gene circuits. Error bars are standard deviation; *N* = 3. Two-sample *t*-test was performed on maximum and minimum mean fluorescence data values. TIP-LITer2.0 ON/OFF had a *P*-value of 2.55E-07 and Deg-LITer2.0 ON/OFF had a *P*-value of 1.02E-08. Kruskal–Wallis test was performed for CV dataset differences. TIP-LITer1.0 versus VVD had a *P*-value of 2.88E-09 and Deg-LITer1.0 versus VVD had a *P*-value of 2.87E-09.

To experimentally increase TetR transcriptional and translational efficiency, we incorporated the P2A sequence to allow polycistronic gene expression under a single promoter (27) (Figure 3A and B). We stably integrated these optogenetic gene circuits termed henceforth TIP-LITer2.0 and Deg-LITer2.0, and sorted cells into monoclonal cultures.

To test the performance of these single-promoter LITer2.0 gene circuits, we studied the same illumination regimes as for the LITer1.0 gene circuits, starting with a light intensity titration with continuous illumination. In agreement with model predictions, the experimental results indicated that increasing TetR transcription and translation efficiency drastically lowered the basal expression by 82.3% and 97.1% for the TIP-LITer2.0 and Deg-LITer2.0 systems, respectively (Supplementary Figures S8F and S3C). Flow cytometry histograms and fluorescence microscopy indicated tight expression control for the two new gene circuits with over 3-fold gene expression induction (Figure 3D–G). Interestingly, the order of basal expression levels flipped compared to the LITer1.0 versions, with the TIP-LITer2.0 having the highest basal expression, while the Deg-LITer2.0 had a similar basal expression to the VVD. Remarkably, despite the massive decrease in basal expression, the CVs of both LITer2.0 gene circuits remained nearly identical to the corresponding LITer1.0 versions, up to 5-fold lower than the VVD system (Figure 3H).

**Figure 4.**
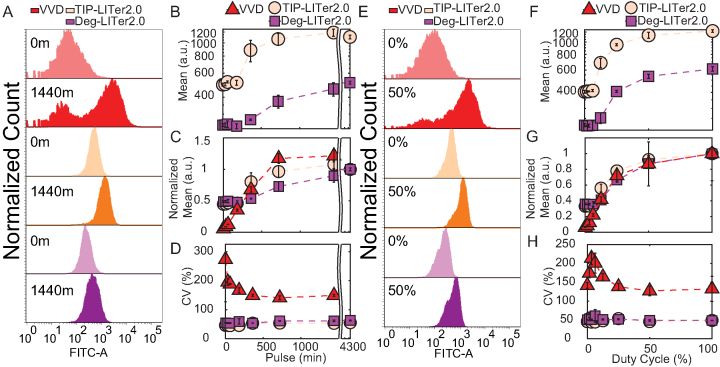
Gene expression control by VVD, TIP-LITer2.0 and Deg-LITer2.0 gene circuits with pulsed illumination. (**A**) Fluorescence histograms for single light pulse length titration. Numbers on histograms represent single pulse durations in minutes. (**B**) Flow cytometry mean fluorescence versus single pulse duration for LITer2.0 gene circuits. (**C**) Normalized mean fluorescence values versus single pulse duration. (**D**) Coefficient of variation (CV) versus single pulse duration. (**E**) Fluorescence histogram distributions for duty cycle experiment. Percentages on histograms indicate duty cycles (fraction of each 1 h period for which light was on). (**F**) Flow cytometry mean fluorescence versus duty cycle for LITer2.0 gene circuits. (**G**) Normalized mean fluorescence values versus duty cycle. (**H**) CV versus duty cycle for all 3 gene circuits. Error bars represent standard deviations; *N* = 3. Two-sample *t*-test was performed on maximum and minimum mean fluorescence data values for pulse and duty-cycle experiments. TIP-LITer2.0 ON/OFF had a *P*-value of 7.72E-06 and Deg-LITer2.0 ON/OFF had a *P*-value of 3.68E-06 for pulse experiment. TIP- LITer2.0 ON/OFF had a *P*-value of 1.01E-06 and Deg-LITer1.0 ON/OFF had a *P*-value of 1.15E-05 for duty-cycle experiments. Kruskal–Wallis test was performed for CV dataset differences. TIP-LITer2.0 versus VVD and Deg-LITer2.0 versus VVD had a *P*-value of 2.88E-09 for the pulse experiment. TIP-LITer2.0 versus VVD had a *P*-value of 2.88E-09 and Deg-LITer2.0 versus VVD had a *P*-value of 2.87E-09 for the duty-cycle experiment.

To test the effects of light pulsing on the LITer2.0 systems, we next examined how single pulse duration affects the mean and noise of gene expression. The LITer2.0 versions had a similar dynamic range to the LITer1.0 systems (Figure 4A–C). Strikingly, the noise remained up to 5.9 times lower for the LITer2.0 gene circuits compared to VVD (Figure 4D). We also analyzed the effect of the duty cycle (Figure 4E–G) and found around 3-fold gene expression induction and up to 4.8-fold noise reduction for the LITer2.0 circuits compared to VVD (Figure 4H).

To test whether the four LITer gene circuits still responded to chemical inducers, we also performed dose–response experiments with the small molecule inducer doxycycline (Supplementary Figure S9). We found higher fold-induction for all LITer gene circuits (16.8, 4.6, 26.1 and 51.2 for TIP-LITer1.0, Deg-LITer1.0, TIP-LITer2.0 and Deg-LITer2.0, respectively). These results imply that future modifications may further improve light-responsiveness of these gene circuits.

Finally, as in the LITer1.0 versions, we inspected the linearity of gene expression versus light intensity by fitting dose–responses of the mean to linear functions (Supplementary Figure S4). The LITer2.0 dose–response was linear up to 225 g.s. from the LPA and its linearity improved compared to the LITer1.0 versions. We attribute this improvement to the increase in transcriptional and translational efficiencies for TetR in the LITer2.0 version, therefore matching GFP production. Whereas linear functions did fit the dose–response data at low induction, they were not the best fit at high induction, where the shape became increasingly non-linear. Consequently, Hill functions better captured the full dose–responses of all gene circuits (Supplementary Figure S10), with the Hill coefficients around 2 for all systems (1.89 [1.46, 2.32], 1.81 [1.5, 2.11], 1.97 [1.62, 2.33] and 2.3 [1.77, 2.83] for the TIP-LITer1.0, Deg-LITer1.0, TIP-LITer2.0 and Deg-LITer2.0, respectively).

Overall, these results show that the LITer2.0 gene circuits are more compact, have lower basal expression, low gene expression noise and a reasonable dynamic range of gene expression induction for various light parameters, making them preferable candidates for precisely controlling expression of a functional gene of interest (GOI).

### Controlling a phenotypically relevant gene with LITer gene circuits

**Figure 5.**
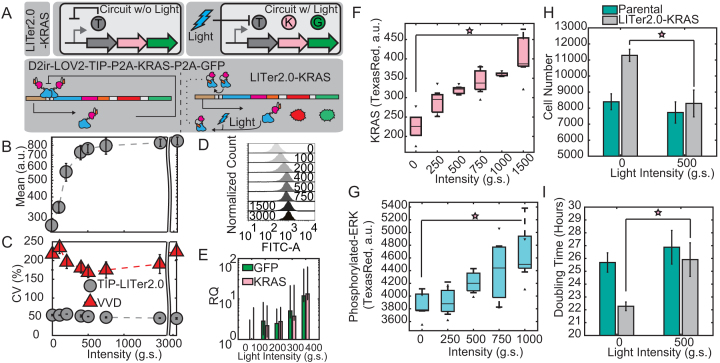
Gene expression control by LITer2.0-KRAS. (**A**) Simplified and detailed schematic of the LITer2.0-KRAS(G12V) gene circuit. ‘T’ is the TetR regulator with TIP, ‘K’ is the KRAS(G12V) protein and ‘G’ is the Reporter output. (**B**) Mean fluorescence intensity dose–response of LITer2.0-KRAS gene circuit with continuous illumination. (**C**) CV dose–response for LITer2.0-KRAS and VVD gene circuits. (**D**) Fluorescence histograms at different light doses with continuous illumination. Numbers on histograms represent light intensities.(**E**) q-RT-PCR measurements of KRAS mRNA levels at increasing doses of light using a log(*y*) axis. (**F**) KRAS protein levels measured by flow cytometry using a fluorescent secondary antibody. (**G**) Phosphorylated-ERK levels measured by flow cytometry with fluorescent secondary antibody. (**H**) Cell growth measured by cell number for the parental (Flp-In 293) cell line and LITer2.0-KRAS cells 72 h after induction. Light causes statistically significant reduction in cell growth compared to basal KRAS expression (uninduced cells), while light has insignificant effects on the parental cell line. Cells were seeded at ∼1200 cells per well. (**I**) Cell growth measured by doubling times for the parental (Flp-In 293) cell line and LITer2.0-KRAS cells 72 h after induction. Light causes statistically significant increase in cell doubling time compared to basal KRAS expression (uninduced cells), while light has insignificant effects on the parental cell line. Experiments were performed with three or four technical replicates. Error bars represent the standard deviation of replicates. Stars indicate statistical significance. Two-sample *t*-test was performed on maximum and minimum mean fluorescence data values. LITer2.0-KRAS ON/OFF had a *P*-value of 3.79E-05. Kruskal–Wallis test was performed for CV dataset differences. LITer2.0-KRAS versus VVD had a *P*-value of 2.88E-09. Two-sample *t*-test was performed on maximum and minimum KRAS and ERK levels giving a *P*-value of 0.0071 and 0.014, respectively. Two-sample *t*-test was performed on cell growth (6H) yielding a *P*-value of 0.0048.

The above results support using LITer systems for controlling gene expression rather than existing, activator-based, feedback-lacking optogenetic systems because NF improves the precision of gene expression control by lowering gene expression noise compared to the latter. For example, if we seek to determine how KRAS expression affects a cellular phenotype, then uniform KRAS expression is beneficial. Based on Figures 1–4, the VVD system could not achieve uniform intermediate expression in all cells. Rather, some cells will have basal expression while other cells will have maximal expression, making it impossible to determine the phenotype for intermediate KRAS levels. On the other hand, the LITer systems enforce uniform, tunable expression, making it possible to answer the above question. Thus, to explore the possibility of using these novel optogenetic NF systems for expressing a functional GOI, we moved toward adapting the TIP-LITer2.0 system to co-express the mutant oncogene KRAS(G12V) with GFP by incorporating an additional P2A sequence. We called the new gene circuit LITer2.0-KRAS (Figure 5A). We built and integrated the system into the Flp-In^TM^ 293 genome and created monoclonal cells lines.

To validate the gene circuit function, we performed flow cytometry for the LITer2.0-KRAS system under an intensity dose–response that indicated up to 3.1-fold gene expression induction for GFP and nearly 5-fold noise reduction versus the VVD system (Figure 5B–D). Furthermore, we also tested the duty cycle-response of the LITer2.0-KRAS system, achieving over 80% expression at 25% duty cycle compared to constant illumination (Supplementary Figure S11). Analyzing the mRNA response of the gene circuit to continuous illumination by qRT-PCR indicated increasing KRAS and GFP mRNA expression (Figure 5E) with over an order of magnitude fold-induction.

KRAS is a major activator of growth factor signaling pathways. Usually, post-translational KRAS activity is considered most relevant for downstream signaling, and most studies focus on KRAS-activating mutations. However, increasing KRAS levels should also lead to higher downstream activity, an effect that has been much less studied. The downstream effector ERK is phosphorylated indirectly by KRAS activity through the intermediaries RAF and MEK. If the LITer2.0-KRAS gene circuit produces increasing levels of active KRAS, this should cause rising phosphorylation of ERK in response to light.

To test the direct and downstream effects of KRAS expression tuning, we measured KRAS mean expression levels and the activity of the downstream effector ERK by immunofluorescence. KRAS levels rose nearly 2-fold due to light induction (Figure 5F), indicating that the gene circuit can induce dose-responsive changes in a functional gene and the reporter simultaneously. Likewise, immunofluorescence indicated a rise in the downstream effector phosphorylated-ERK in response to light, confirming that precisely controlled KRAS levels affect downstream signaling (Figure 5G).

Finally, we checked whether the dose-responsive increases in KRAS levels, and therefore phosphorylated-ERK, could affect cell proliferation as previously demonstrated (54,55). We grew cells for ∼72 h under various light conditions. Afterwards, we stained cells with NucBlue™ and imaged them by microscopy to count their total number per field. Cell growth decreased with increasing light exposure for LITer2.0-KRAS cells, while light did not affect the growth of parental cells significantly (Figure 5H and Supplementary Figure S12). Interestingly, we observed that the uninduced basal expression for the LITer2.0-KRAS gene circuit provides some benefit in cell growth compared to parental cells. This indicates that an optimal, low KRAS level may maximize cell growth, while higher Kras may lead to senescence (56). Accordingly, converting the cell number into doubling time (Figure 5I) indicated faster growth under low overexpression of KRAS and doubling times reminiscent of parental cells at intermediate doses of light. Finally, to validate that the observed effects were due to KRAS induction and not light alone, we also analyzed KRAS and phosphorylated-ERK response to doxycycline that recapitulated the light-controlled KRAS findings (Supplementary Figure S13).

Overall, the LITer2.0 circuits are beneficial for precision control of intermediate gene expression levels, while maintaining low gene expression noise for a functional GOI, and enabling future studies for single-cell control.

## DISCUSSION

Optogenetic gene expression control in mammalian cells has relied on transient transfection and on light-inducible transcriptional activators. Therefore, the precision of these systems is currently unknown. Negative feedback regulation is a wide-spread mechanism of noise reduction in nature and synthetic biology, but it requires repressors rather than activators. This is possibly the reason why negative feedback in optogenetic gene expression systems has only been achieved by *in silico* control, using computers for automatic light intensity adjustments according to the expression of continuously monitored cultured microbial cells (30,31). This type of *in silico* control requires expensive computational and optical setups, and it is not ideal for controlling single cells that move and divide. The possibility of genetic negative feedback regulation by *in vitro* control has been unexplored, especially in mammalian cells.

Here, we describe five novel optogenetic gene circuits that can be utilized over various light parameters for controlling gene expression levels and reducing noise, enabling single-cell control systems.

We report that all LITer synthetic gene circuits can drastically reduce noise compared to benchmark tools such as LightOn (28), facilitating more precise control of gene expression in single cells in the future. The stable monoclonal cells lines we developed or similar ones can be utilized for exploring functional effects of gene expression levels and noise in the RAS or other pathways, or various phenotypically relevant genes in future single-cell investigations.

Given the effects of various light exposure modes, periodic pulsing may be an optimal mode for controlling optogenetic gene circuits for at least two reasons. First, the duty cycle can minimize the time cells are exposed to light, preventing unnecessary phototoxicity. Second, relatively modest duty cycles can achieve expression levels approaching those for continuous illumination, and for the VVD system, they can even surpass it.

We show that all the systems remain responsive to chemical induction with up to 51-fold change between ON/OFF states, implying that there is ample room to improve circuit architectures for enhanced responses with light. Specifically, we envision these tools can be improved by probing additional peptides with higher affinities, more efficient LOV2 domains and more efficient degradation tags. We also expect further modeling will lead to architecture manipulations that can achieve a wider dose–response to light, lower basal expression, lower noise and higher fold-change.

Finally, the gene circuit components we present here (including small peptides and degradation tags that can be synthesized *in vitro*) offer a platform for modifying existing Tet-based systems into light-responsive tools for precisely controlling single-cells. This will allow spatiotemporal control that has remained cumbersome and restricted by chemical induction alone. Future objectives of this work will aim at scaling the feedback systems to libraries of GOIs and perturbing these GOIs in a spatially restricted manner with single-cell resolution. Such regulated systems provide an ideal platform for producing accurate and precise gene expression compared to unregulated gene circuits that can achieve accurate expression but have wider gene expression noise and therefore less precision. We also predict that TIP, degradation tags and other peptides will offer a general-purpose platform that can be utilized for expansion to other common gene circuit architectures including positive regulation, negative regulation and positive feedback that depend on TetR and rtTA proteins.

## DATA AVAILABILITY

- Plasmid sequence files can be obtained here: https://www.addgene.org/Gabor_Balazsi/.
- Flow cytometry data, modeling and microscopy data can be found here: https://openwetware.org/wiki/CHIP:Data.

## Supplementary Material

gkz556_Supplemental_File
