# Supplementary material for: Noise-reducing optogenetic negative-feedback gene circuits in human cells
Source: Nucleic Acids Res. 2019 Jul 3;47(14):7703–14. doi: 10.1093/nar/gkz556 (PMC6698750; doi:10.1093/nar/gkz556)
Supplement: gkz556_Supplemental_File [file gkz556_supplemental_file.pdf]

Noise-Reducing Optogenetic Negative-Feedback Gene Circuits in Human Cells

Supplementary Information

Michael Tyler Guinn[1,2,3] and Gábor Balázsi[1,2,*]

[1]Biomedical Engineering Department, Stony Brook University, Stony Brook, NY 11974 USA

[2]Laufer Center for Physical and Quantitative Biology, Stony Brook University, Stony Brook, NY 11794 USA

[3]Stony Brook Medical Scientist Training Program, 101 Nicolls Road, Stony Brook, NY 11794 USA

Keywords: Synthetic biology, optogenetics, light-inducible, noise, feedback

[*]Corresponding author
Gábor Balázsi, Ph.D.
Henry Laufer Associate Professor
Laufer Center for Physical & Quantitative Biology
115C Laufer Center, Z-5252
Stony Brook University
Stony Brook, NY 11794
Tel. +1-631-632-5414;
Email: gabor.balazsi@stonybrook.edu

Table of Contents

Molecular Cloning: 3
Plasmid Construction: 3
Plasmid Maps (SnapGene ®): 5
Stochastic Modeling: 13
Chemical Reactions: LITer1.0 Systems 13
Chemical Reactions: LITer2.0 Systems 14
Deterministic Models 15
Hill Function Fitting: 17
Linearity Assessment 18
Supporting Figures: 19
Supporting Tables: 32
Supporting References: 36

Molecular Cloning:

Plasmid Construction:

CMV-TetR-LOV-TIP (pMTG01-Amp-FRT): The hTetR::NLS:EGFP was digested from pDN-D2ir-TNG4kwh[1] and cloned into pcDNA5/FRT (Thermo Fisher Scientific, V652020) using Spe & Sph restriction enzyme sites. This produced pDN-D2irTN2aG5kwh (pTG01). The hTetR fragment was digested from pDN-D2irTN6kwh[1] and cloned into pTG01 using KasI and NotI restriction enzyme sites. This produced vector pCB-D2irTN5kwh (pTG02). The vector pKH546 (pTG03) was a gift from Professor Wilfried Weber[2]. The hTetR sequence was PCR amplified from pTG02 using primers P1 and P2. The LOV-TIP sequence was PCR amplified from pTG03 using primers P3 and P4. The two fragments were combined in overlap PCR and cloned into pTG01 using BamHI & AgeI restriction enzyme sites. This produced CMV-TetR-LOV-TIP vector. The TetR-LOV2 fusion protein was connected with a ASGAGA peptide linker.

CMV-TetR-LOV-RRRG-Degron (pMTG02-Amp-FRT): The vector pKH546 (pTG03) was a gift from Professor Wilfried Weber[2]. The hTetR sequence was PCR amplified from pTG02 using primers P1 and P2. The LOV-RRRG sequence was PCR amplified from pTG03 using primers P3 and P5. The two fragments were combined in overlap PCR and cloned into pTG01 using BamHI & AgeI restriction enzyme sites. This produced CMV-TetR-LOV-RRRG(Degron) vector. The TetR-LOV2 fusion protein was connected with a ASGAGA peptide linker.

TIP-LITer1.0 or CMV-TetOx2-TetR-LOV-TIP---CMV-TetOx2-GFP (pMTG03-Amp-FRT): The CMV-GFP fragment was digested from pDN-D2irG4kwh[1] and cloned into pTG02 using NotI & KasI restriction enzyme sites. This produced pCB-D2ir-GKwh-D2ir-TN5kwh (pTG04). The TetR-LOV-TIP fragment was digested from pMTG01-Amp-FRT and cloned into pTG04 using MluI & EcoRI restriction enzyme sites. This produced the Dual-NF-TIP or CMV-TetOx2-TetR-LOV-TIP---CMV-TetOx2-GFP vector.

Deg-LITer1.0 or CMV-TetOx2-TetR-LOV-RRRG---CMV-TetOx2-GFP (pMTG04-Amp-FRT): The TetR-LOV-RRRG fragment was digested from pMTG01-Amp-FRT and cloned into TG04 using MluI & EcoRI restriction enzyme sites. This produced the Deg-LITer1.0 or CMV-TetOx2-TetR-LOV-RRRG---CMV-TetOx2-GFP vector.

TIP-LITer2.0 or CMV-TetOx2-TetR-LOV-TIP-P2A-GFP (pMTG05-Amp-FRT): The hTetR-LOV-TIP sequence was PCR amplified from pMTG03-Amp-FRT using primers P6 and P7. The P2A-GFP-Backbone sequence was PCR amplified from pTG01 using primers P8 and P9. The fragments were combined using NEBuilder HiFi DNA Assembly

Reaction Protocol (catalog number: E2621S). This produced the TIP-LITer2.0 or CMV-TetOx2-TetR-LOV-TIP-P2A-GFP.

Deg-LITer2.0 or CMV-TetOx2-TetR-LOV-RRRG-P2A-GFP (pMTG06-Amp-FRT): The hTetR-LOV-RRRG sequence was PCR amplified from pMTG04-Amp-FRT using primers P6 and P10. The P2A-GFP-Backbone sequence was PCR amplified from pTG01 using primers P8 and P9. The fragments were combined using NEBuilder HiFi DNA Assembly Reaction Protocol (catalog number: E2621S). This produced the Deg-LITer2.0 or CMV-TetOx2-TetR-LOV-RRRG-P2A-GFP vector.

VVD system or 5xUAS-mNeonGreen--CMV-Gal4-VVD-p65 (pMTG07-Amp-FRT): The vector pGAVPO (pTG05) was a gift from Professor Yi Yang[3]. The vector HIV-pBOB-CAG-mNeonGreen-2xNLS (pTG06) was a gift from Gerald Pao[4]. The vector pU5-Gluc (pTG07) was a gift from Professor Yi Yang[3]. The mNeonGreen sequence was PCR amplified from pTG06 using primers P11 and P12 and cloned into pTG07 using HindIII and ApaI restriction enzyme sites. This produced 5xUAS-mNeonGreen-NLSx2 (pTG08) vector. The FRT-Backbone sequence was PCR amplified from pTG01 using primers P13 and P14. The 5xUAS-mNeonGreen-NLSx2 sequence was PCR amplified from pTG08 using primers P15 and P16. The CMV-Gal4-VVD-p65 sequence was PCR amplified from pTG05 using primers P17 and P18. The fragments were combined using NEBuilder HiFi DNA Assembly Reaction Protocol (catalog number: E2621S). This produced the 5xUAS-mNeonGreen--CMV-Gal4-VVD-p65 vector.

TIP-LITer2.0-KRAS(G12V) or CMV-TetOx2-TetR-LOV-TIP-P2A-KRAS(G12V)-P2A-GFP (pMTG8-Amp-FRT): The vector pEGFP-C3-KRAS4B-G12V (pTG09) was a gift from Dr. Mark Philips[5]. The KRAS4B (G12V) sequence was PCR amplified from pTG09 using primers P41 and P42. The P2A-GFP sequence was PCR amplified from pMTG05-Amp-FRT using primers P43 and P44. The Backbone-CMV-TetR-LOV-TIP-P2A sequence was PCR amplified from pMTG05-Amp-FRT using primers P45 and P46. The fragments were combined using NEBuilder HiFi DNA Assembly Reaction Protocol (catalog number: E2621S). This produced the CMV-TetOx2-TetR-LOV-TIP-P2A-KRAS(G12V)-P2A-GFP vector (pMTG8-Amp-FRT).

Plasmid Maps (SnapGene ®):

pMTG01: CMV-TetR-LOV-TIP

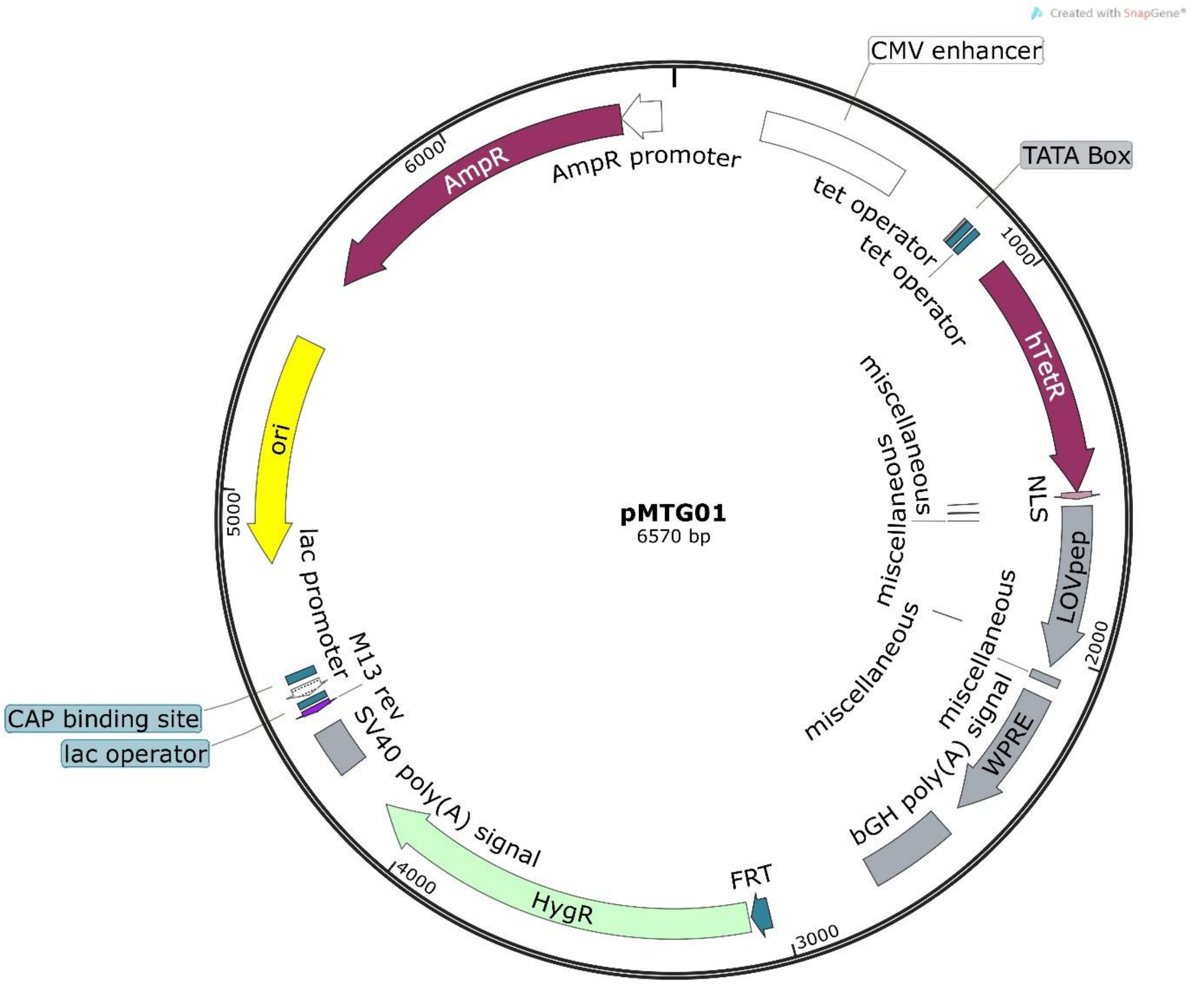

pMTG02: CMV-TetR-LOV-RRRG-Degron

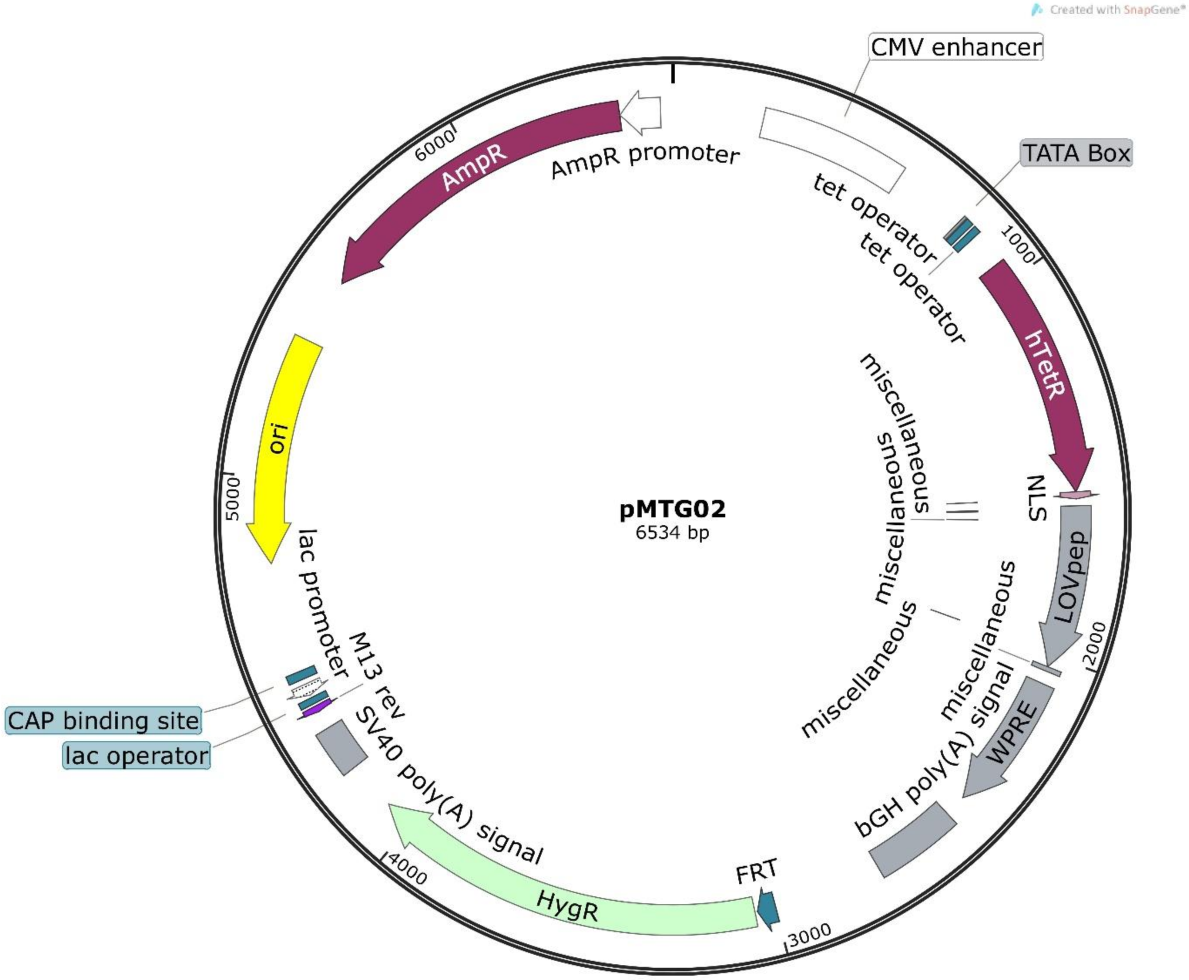

pMTG03: TIP-LITer1.0 or CMV-TetOx2-TetR-LOV-TIP---CMV-TetOx2-GFP

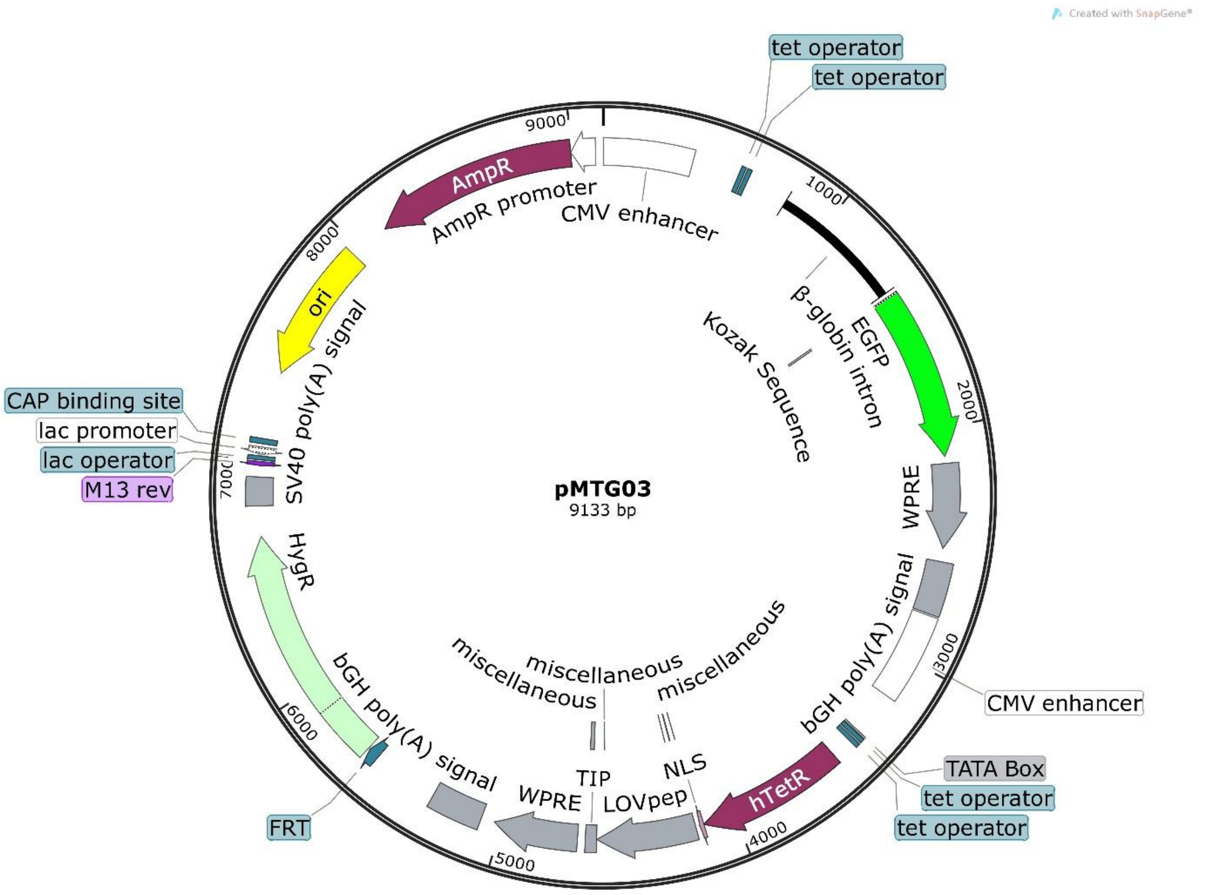

pMTG04: Deg-LITer1.0 or CMV-TetOx2-TetR-LOV-RRRG---CMV-TetOx2-GFP

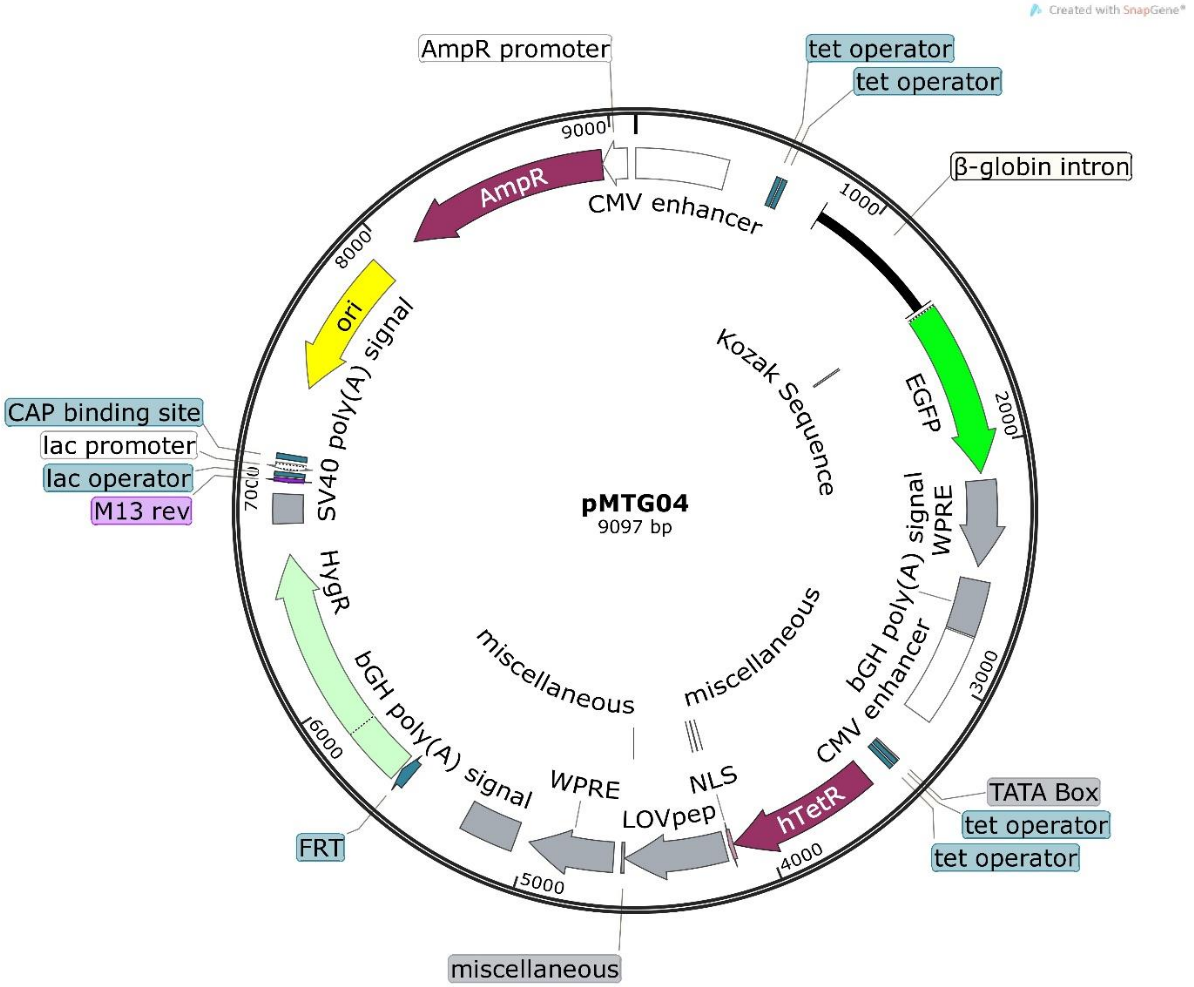

pMTG05: TIP-LITer2.0 or CMV-TetOx2-TetR-LOV-TIP-P2A-GFP

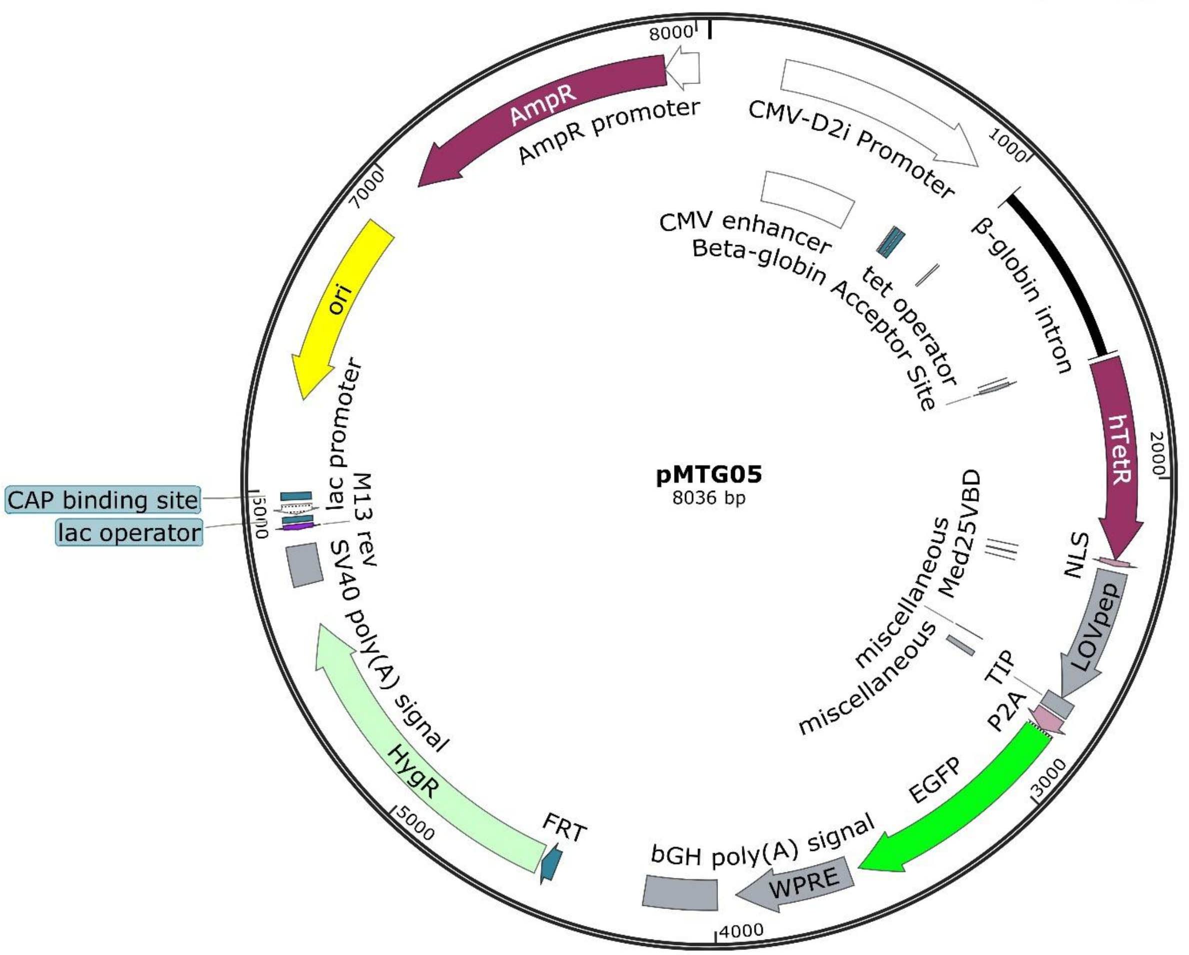

pMTG06: Deg-LITer2.0 or CMV-TetOx2-TetR-LOV-RRRG-P2A-GFP

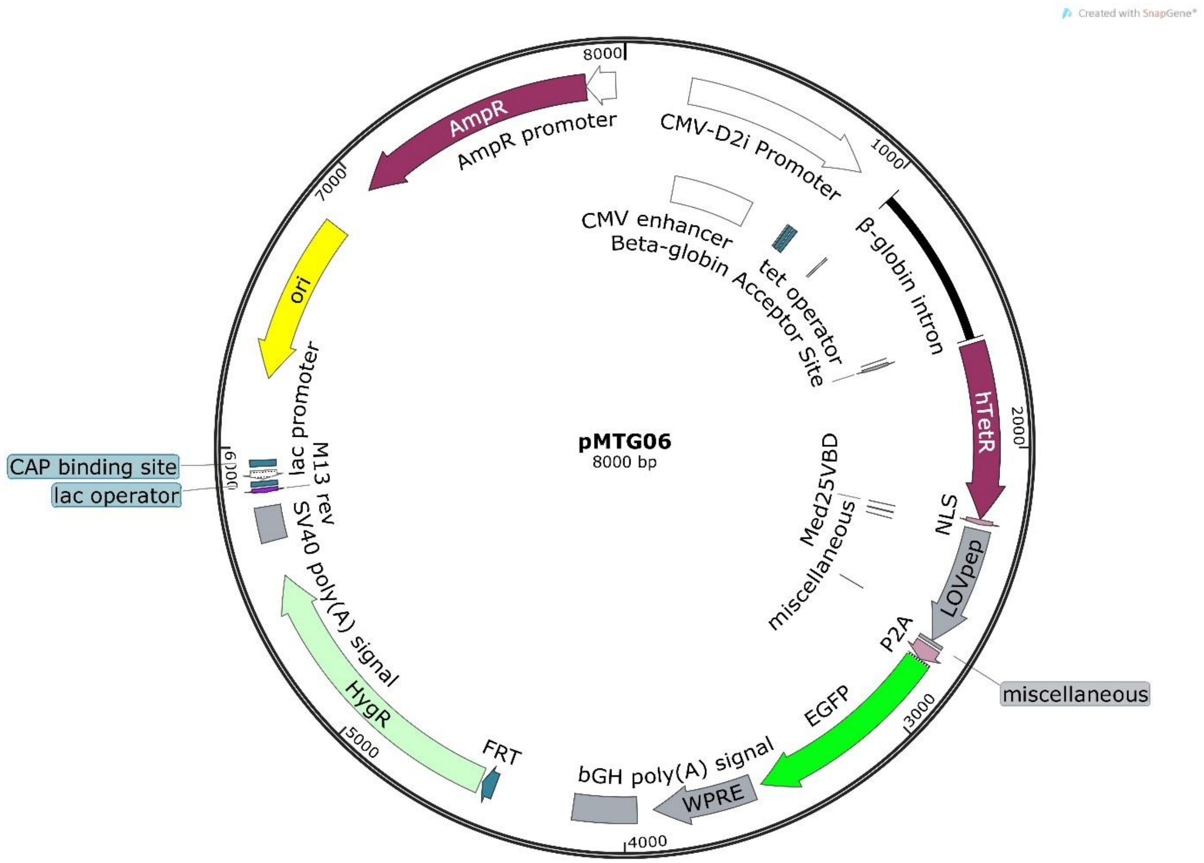

pMTG07: VVD system or 5xUAS-mNeonGreen--CMV-Gal4-VVD-p65

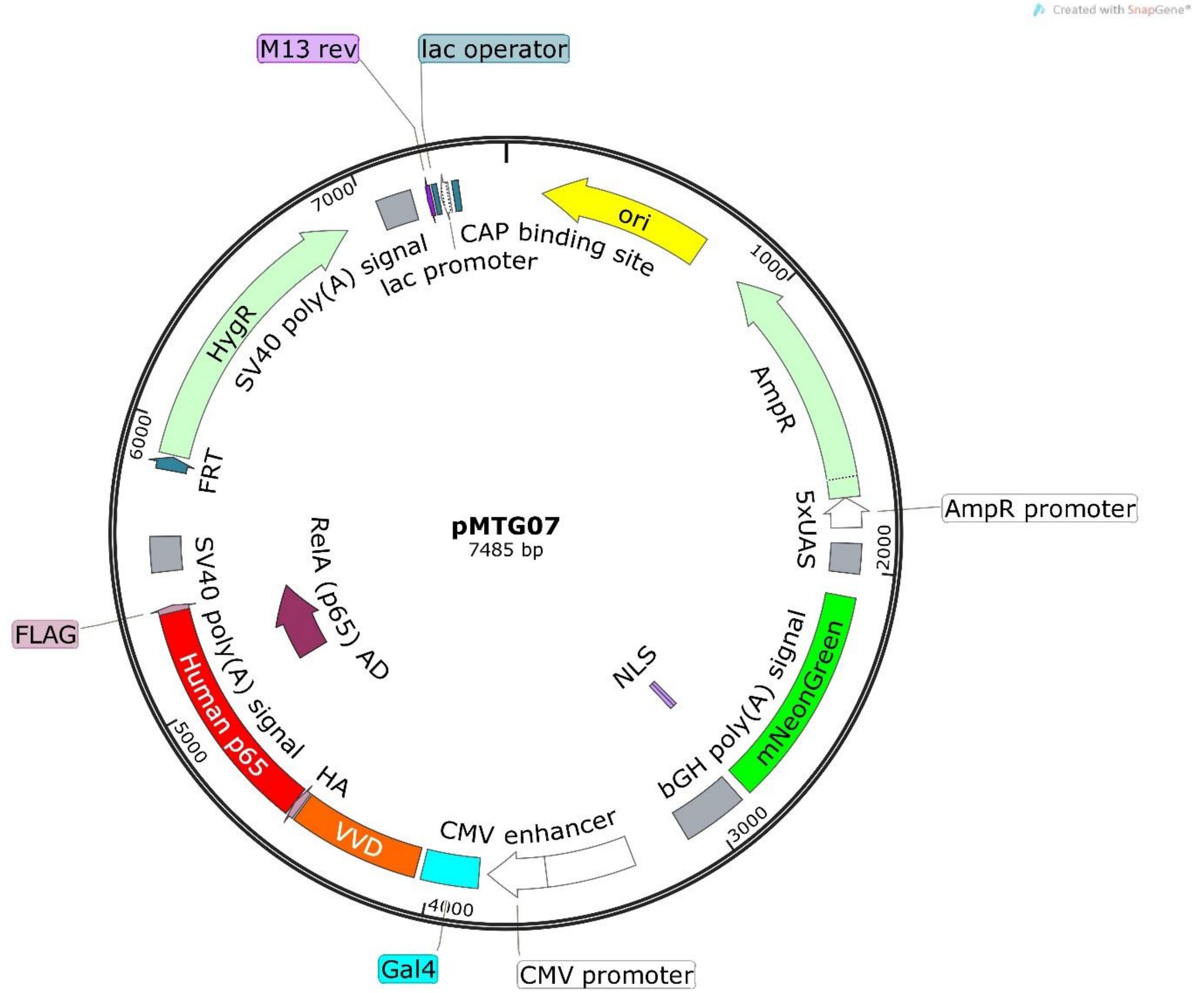

pMTG08: TIP-LITer2.0-KRAS(G12V) or CMV-TetOx2-TetR-LOV-TIP-P2A-KRAS(G12V)-P2A-GFP

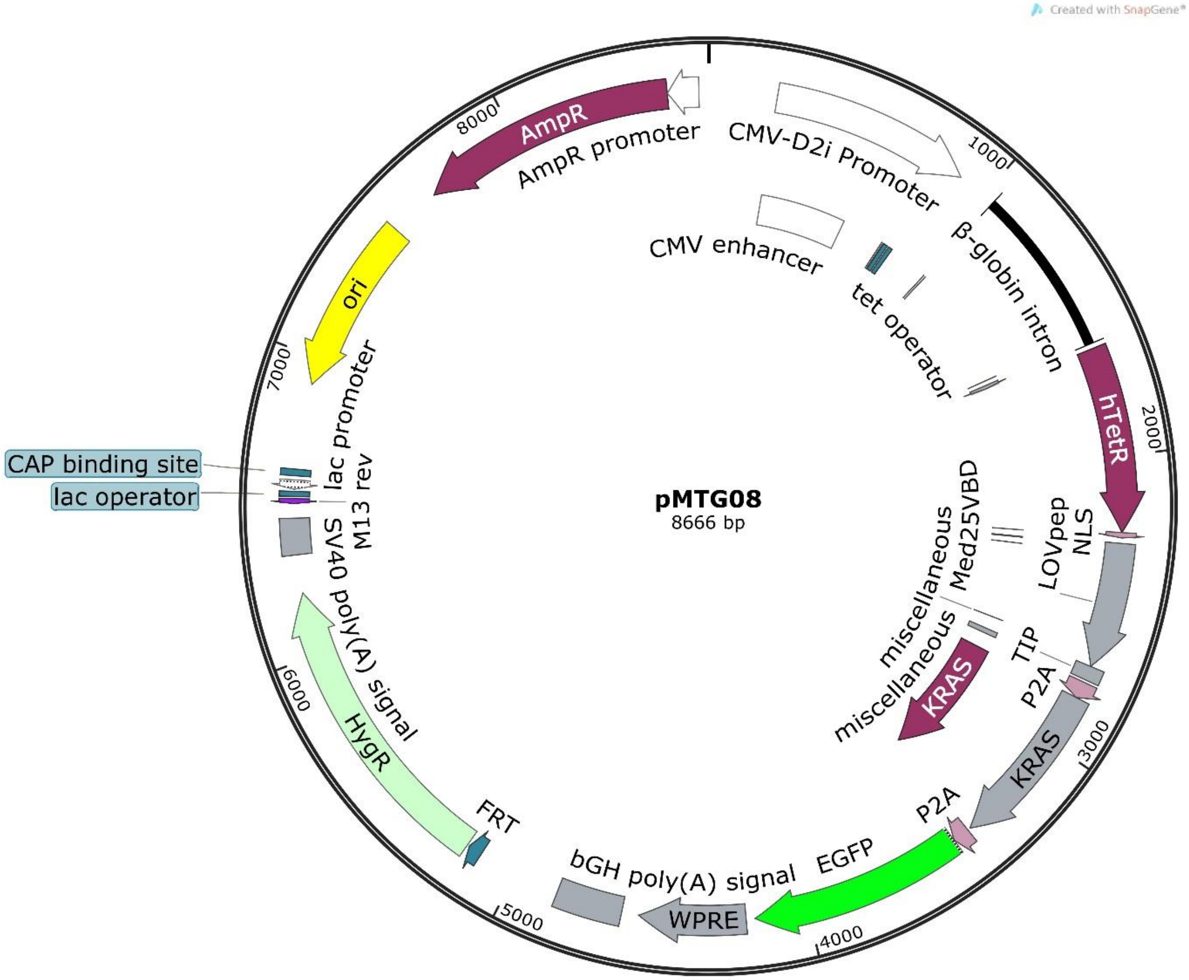

Stochastic Modeling:

Chemical Reactions: LITer1.0 Systems

$P00 \xrightarrow{m_R} P00 + M_R$	Free TetR promoter making TetR mRNA
$P0 \xrightarrow{m_G} P0 + M_G$	Free GFP promoter making GFP mRNA
$\emptyset \xrightarrow{l_R} M_R$	Basal expression of TetR promoter
$\emptyset \xrightarrow{l_G} M_G$	Basal expression of GFP promoter
$M_R \xrightarrow{g_1} \emptyset$	TetR mRNA degraded
$M_G \xrightarrow{g_1} \emptyset$	GFP mRNA degraded
$M_R \xrightarrow{t_R} M_R + R$	TetR mRNA translation
$M_G \xrightarrow{t_G} M_G + G$	GFP mRNA translation
$R \xrightarrow{g_2} \emptyset$	TetR protein degraded
$G \xrightarrow{g_2} \emptyset$	GFP protein degraded
$R + P00 \xrightarrow{b} P01$	TetR binding unbound TetR promoter
$P01 \xrightarrow{u} P00 + R$	TetR unbinding single-bound TetR promoter
$R + P01 \xrightarrow{b} P02$	TetR binding single-bound TetR promoter
$P02 \xrightarrow{u} P01 + R$	TetR unbinding double-bound TetR promoter
$R + P0 \xrightarrow{b} P1$	TetR binding unbound GFP promoter
$P1 \xrightarrow{u} P0 + R$	TetR unbinding single-bound GFP promoter
$R + P1 \xrightarrow{b} P2$	TetR binding single-bound GFP promoter
$P2 \xrightarrow{u} P1 + R$	TetR unbinding double-bound GFP promoter

Species in **Supporting Table 2**

Chemical Reactions: LITer2.0 Systems

$P0 \xrightarrow{m_{RG}} P0 + M_{RG}$	Free TetR promoter making TetR/GFP mRNA
$\emptyset \xrightarrow{l_{RG}} M_{RG}$	Basal expression of TetR/GFP promoter
$M_{RG} \xrightarrow{g_1} \emptyset$	TetR/GFP mRNA degradation
$M_{RG} \xrightarrow{t_{RG}} M_{RG} + R + G$	TetR/GFP mRNA translation
$R \xrightarrow{g_2} \emptyset$	TetR protein degradation
$G \xrightarrow{g_2} \emptyset$	GFP protein degradation
$R + P0 \xrightarrow{b} P1$	TetR binding unbound TetR/GFP promoter
$P1 \xrightarrow{u} P0 + R$	TetR unbinding single-bound TetR/GFP promoter
$R + P1 \xrightarrow{b} P2$	TetR binding single-bound TetR/GFP promoter
$P2 \xrightarrow{b} P1 + R$	TetR unbinding double-bound TetR/GFP promoter

Species in **Supporting Table 2**

Deterministic Models

The LITer system can be represented by the reaction scheme shown below (using the Degron-based LITer).

Light-activation of the LOV2 domain is at the core of the LITer system. This includes repressor (R)and degron-activated repressor (U). A simplifying step is to assume that light converts the repressor directly into the degron-activted, repressor U.

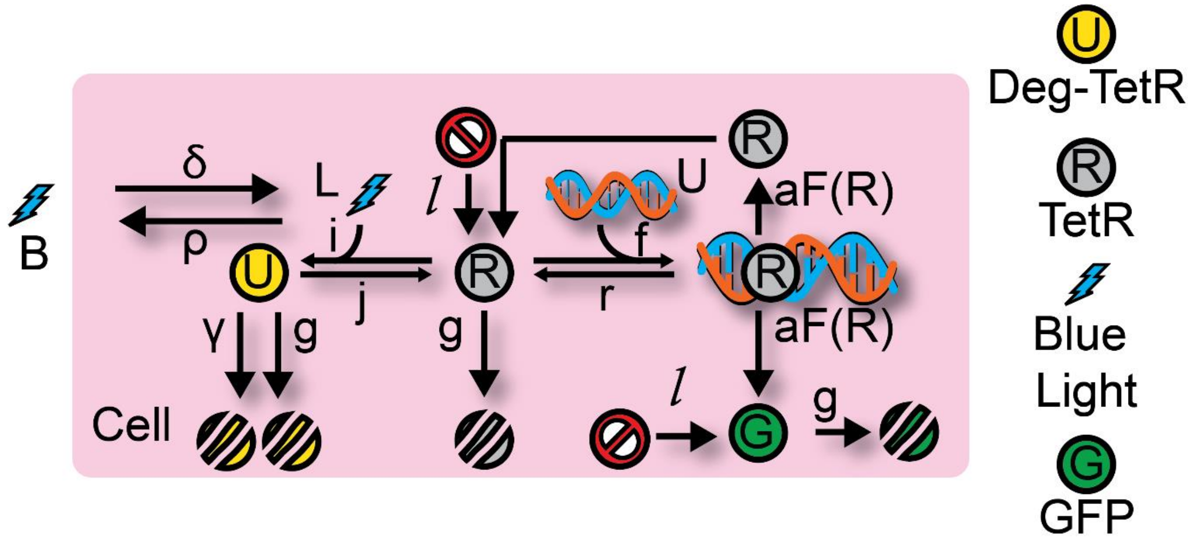

The reactions and ordinary differential equations (ODEs) for this system are:

Symbols:

R: $TetR\ repressor$

U: $degron\ active\ TetR\ repressor$

G: GFP

B: $Extracellular\ light$

L: $Intracellular\ light$

Chemical Equations:

$B \xrightarrow{\delta} L \xrightarrow{\rho} \emptyset$; transfer of light into cell & loss of light

$\emptyset \xrightarrow{aF(R)} R$; synthesis of TetR

$\emptyset \xrightarrow{aF(R)} G$; synthesis of GFP

$R \xrightarrow{iL} U$; LOV2 activation

$U \xrightarrow{j} R$; LOV2 inactivation

$R \xrightarrow{g} \emptyset$; degradation of TetR

$G \xrightarrow{g} \emptyset$; degradation of GFP

$U \xrightarrow{\gamma+g} \emptyset$; degradation of Light active TetR

<u>Differential Equations:</u>

$\dot{R} = aF(R) - iLR - gR + jU + l$

$\dot{U} = iLR - jU - (\gamma + g)U$

$\dot{G} = aF(R) - gG + l$

$\dot{L} = \delta B - iLR - \rho L$

This model deviates from the standard Linearizer model in the basal expression term l and the refolding term j, both of which were 0 for the standard model.

The effect of adjusting these parameters away from the standard linearizer can be seen in Supporting Figure 6.

Hill Function Fitting:

To further inspect gene circuits dose responses, we fit a Hill function to light dose-response data (SE1, Supplement Figure 8).

$$F_x(x) = \frac{ax^b}{c^b + x^b} + d \qquad \text{[SE1]}$$

The ‘a’ parameter represents the theoretical maximum gene expression of the system, the ‘b’ parameter is the Hill coefficient, the ‘c’ parameter is the light value at half-induction, the ‘d’ parameter is the leakiness of the systems, and ‘x’ is the light concentration. We analyzed parameter differences from the Hill function between the different gene circuits (Supporting Table 4). We show the values and confidence intervals as well for all parameters (Supporting Figure 8) which shows all values are statistically significant.

Analyzing all feedback architectures, we found R^2 values around 0.98 or 0.99 for all systems involved. Additionally, when we plotted the 99% confidence interval for the experimental data, fitting the Hill function, we found extremely tight fitting, capturing all data points involved. Overall, these results point towards a characterization of the gene circuit dose-responses and allow end-point users to replace reporters with or add functional GOIs for probing endogenous biological questions.

Linearity Assessment

Previously, negative feedback and identical promoters were found to produce linearization of the dose response[1,6]. Linearity depended on inducer binding to the repressor, as the main source of decay for repressor and inducer below saturation. This implies that the dilution and degradation of inducer and repressor are relatively small compared to binding of the two into an inducer-repressor complex.

Additionally, non-identical promoters caused deviation of linearity in negative feedback systems[6]. For example, when the promoter driving a reporter gene and a promoter driving a repressor gene differed in the number of Tet operator sites, there was lower linearity between circuit input and output. This contrasted when the promoters and operators were identical, which increased linearity.

In the LITer1.0 system, promoters were identical, however there was an intron and Kozak sequence for the GFP, but not the TetR. This likely altered transcriptional and translational responses to promoter states, causing lower linearity than the original chemical linearizers. Additionally, another source of lower linearity in the LITer systems might be light indirectly affecting TetR through activation of TIP or a degradation tag, while doxycycline directly acts on TetR. There is most likely lower affinity for TIP than the small molecule doxycycline and the light-induced degradation is probably less effective that doxycycline inhibition, lowering linearity from previously observed.

To seek improvements of linearity, we constructed the LITer2.0 systems, where we found higher linearity for both the TIP-LITer2.0 system and Deg-LITer2.0 systems (Supporting Figure S4). The most linear range of expression in response to light was between 75 and 225 g.s. on the LPA device shown below.

Supporting Figures:

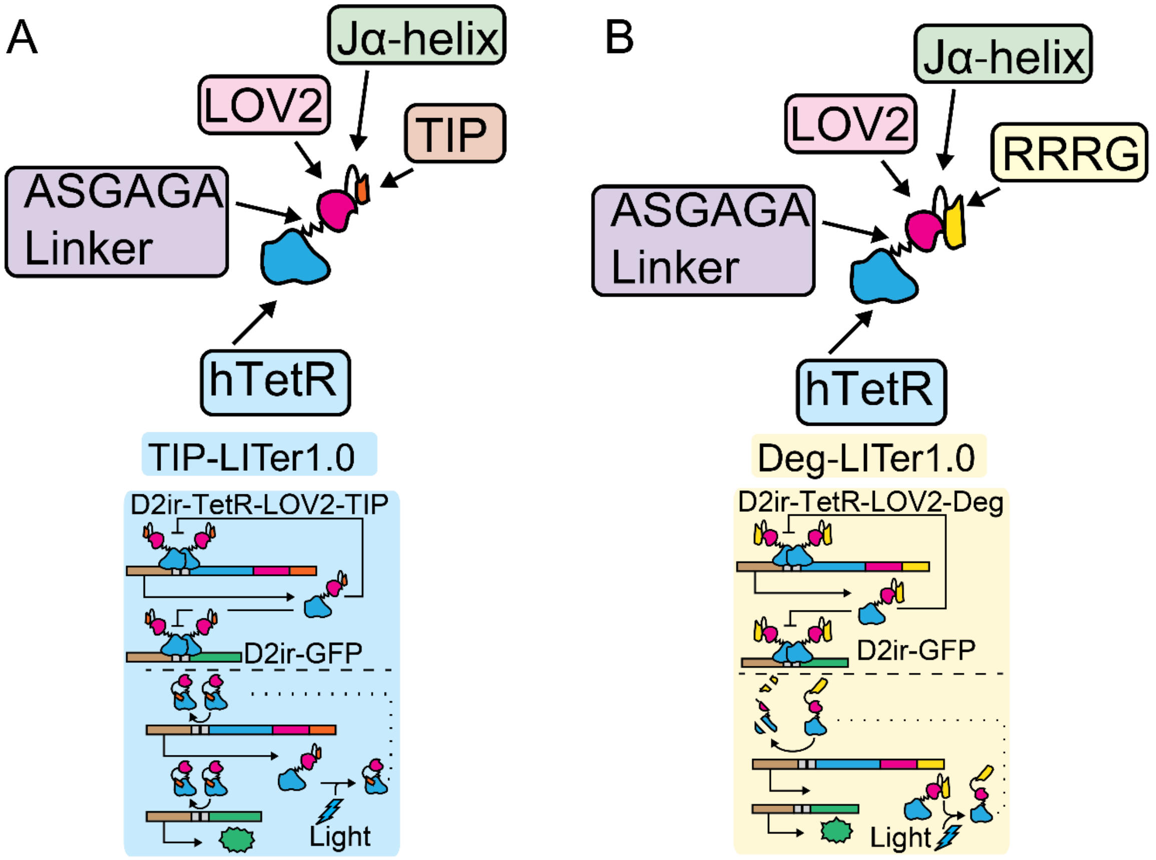

Supporting Figure S1: Genetic architectures of fusion proteins. (A) Fusion protein used in the TIP-LITer1.0 & TIP-LITer2.0 gene circuits. (B) Fusion protein used in the Deg-LITer1.0 & Deg-LITer2.0 gene circuits.

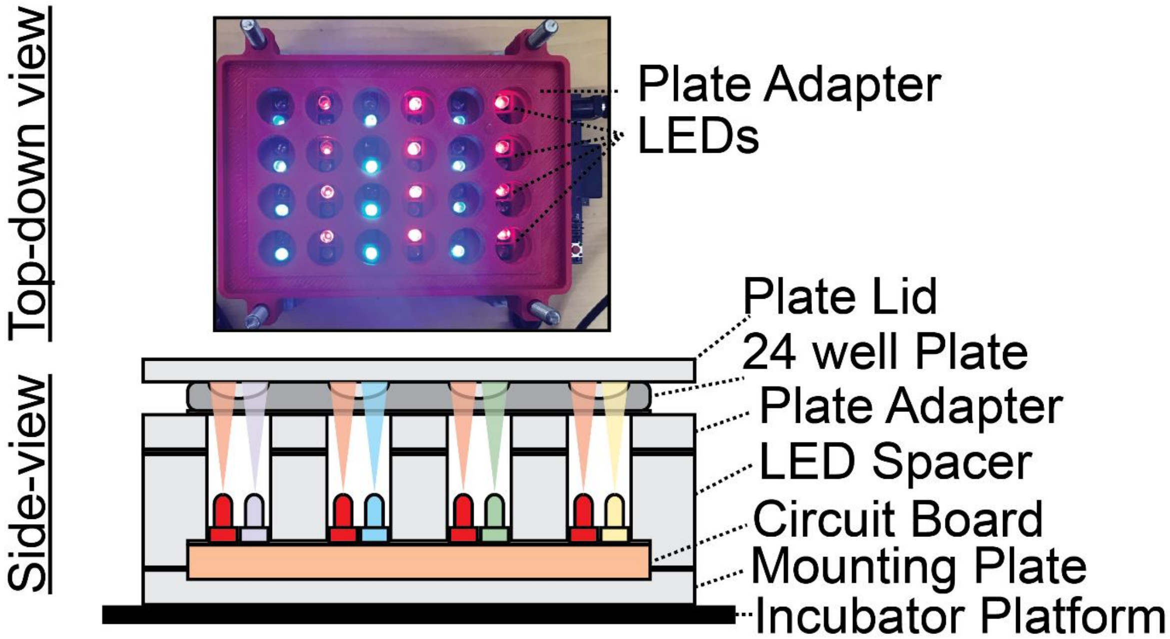

Supporting Figure S2: Light-Induction Equipment. The Light Plate Apparatus can contain a mammalian cell culture plate and consists of 24 wells, each of which has two LEDs that can be used for tuning two different wavelengths. Each LED can be turned on continuously, pulsed, or used in complex patterns[7] to induce gene circuit expression outputs.

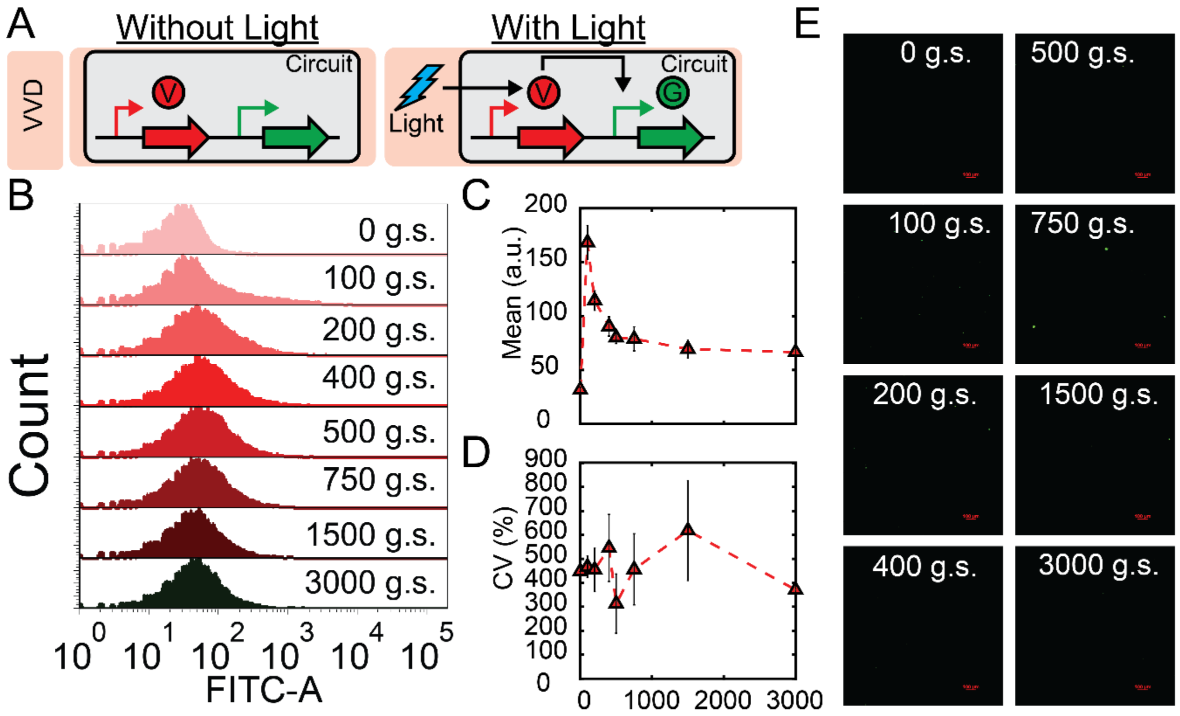

Supporting Figure S3: Effect of prolonged VVD gene circuit exposure to blue light (72h). (A) Schematic illustration of VVD gene circuit stably integrated within Flp-In cell genome. (B) Population histogram distributions for flow cytometry performed 72h post light-induction. (C) Flow cytometry mean fluorescence expression over light intensity titration. (D) Coefficient of variation (CV) dose response of gene circuit. (E) Fluorescence microscopy of cells over light intensity titration.

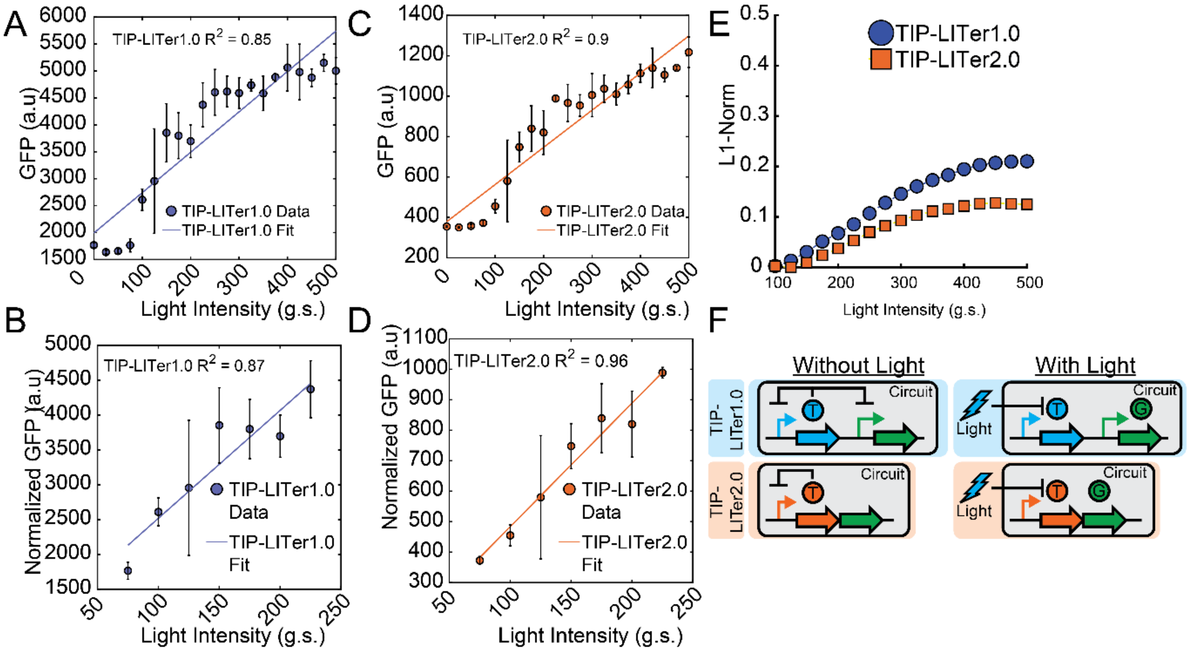

Supporting Figure S4: Gene circuit linearity & fold-induction for TIP-LITer1.0 & TIP-LITer2.0. (A) Wide Intensity dose response assessed for TIP-LITer1.0. (B) Optimal linear range for TIP-LITer1.0 between 75-225 g.s. (C) Wide Intensity dose response assessed for TIP-LITer2.0. (D) Optimal linear range for TIP-LITer2.0 between 75-225 g.s. (E) L1-norm for Intensity dose response for TIP-LITer1.0 & TIP-LITer2.0. (F) Schematic illustration for TIP-LITer1.0 & TIP-LITer2.0.

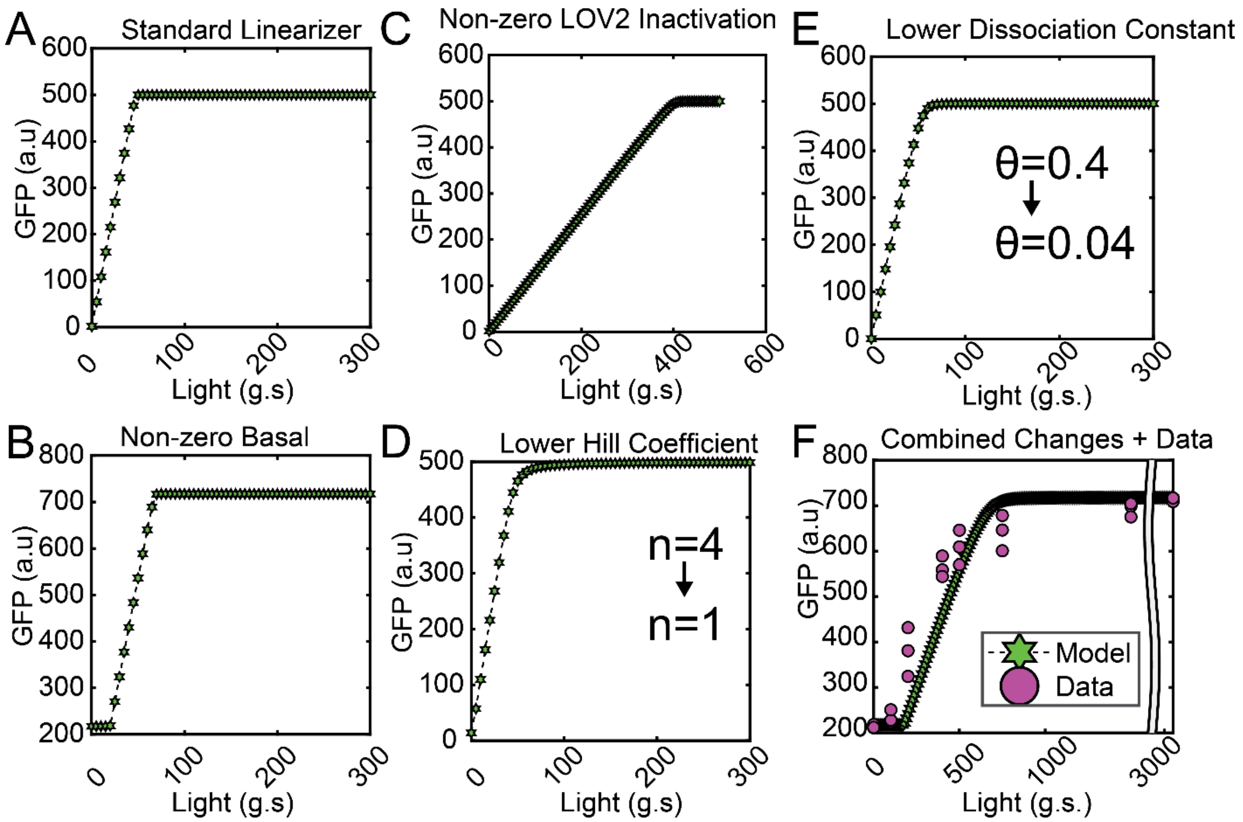

Supporting Figure S5: Adjustments to the standard linearizer model[1]. (A) Standard linearizer model adjusted for light in place of the small chemical doxycycline. (B) Standard linearizer model adjusted to have non-zero basal expression which matches experimental results. (C) Standard linearizer model adjusted to incorporate LOV2 refolding to inactive state, thereby increasing the light needed to induce a level of GFP. (D) Standard linearizer model adjusted to have lower Hill coefficient that results in decreased sharpness of the linear range when approaching saturation. (E) Standard linearizer model adjusted to have lower dissociation constant that results in decreased sharpness of the linear range with approaching saturation. (F) Standard linearizer model adjusted to incorporate all parameters within panels A-E with experimental data from Deg-LITer2.0.

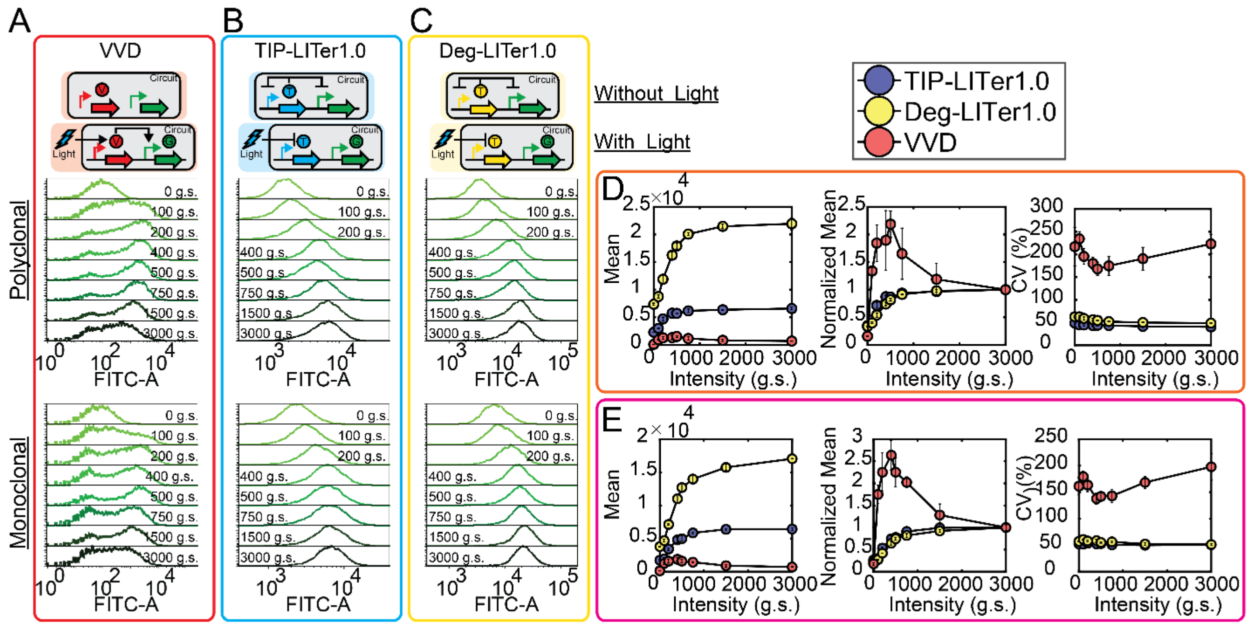

Supporting Figure S6. Monoclonal and polyclonal populations. (A) Schematic illustration of VVD gene circuit and flow cytometry data for polyclonal light dose-response as well as monoclonal light dose-response. (B) Schematic illustration of TIP-LITer1.0 gene circuit and flow cytometry data for polyclonal light dose-response as well as monoclonal light dose-response. (C) Schematic illustration of Deg-LITer1.0 gene circuit and flow cytometry data for polyclonal light dose-response as well as monoclonal light dose-response. (D) Raw mean, normalized mean, & noise profile of polyclonal VVD, TIP-LITer1.0, & Deg-LITer1.0 gene circuits. (E) Raw mean, normalized mean, & noise profile of monoclonal VVD, TIP-LITer1.0, & Deg-LITer1.0 gene circuits.

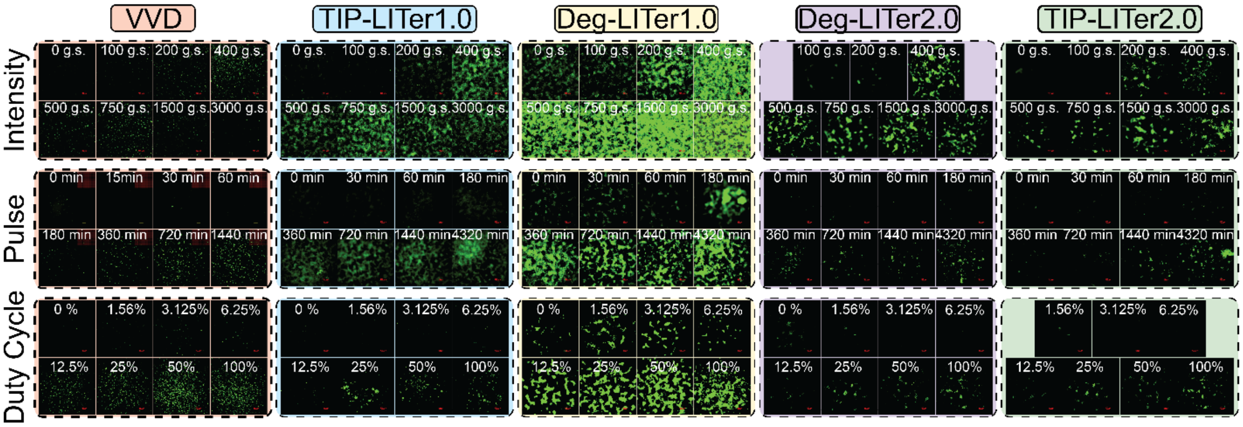

Supporting Figure S7: Gene circuit dose responses by fluorescence microscopy. Florescence microscopy from five cell lines integrated with various gene circuits under various experimental conditions including intensity dose response, pulse dose response, and duty cycle dose response. Each grouped column contains a single cell-line of interest with the corresponding gene circuit listed above, while the rows correspond to the light parameter being explored. All cells were exposed to the same exposure time and gain within a given condition (e.g. VVD intensity dose response from 0 g.s. to 3000 g.s.) and normalized accordingly.

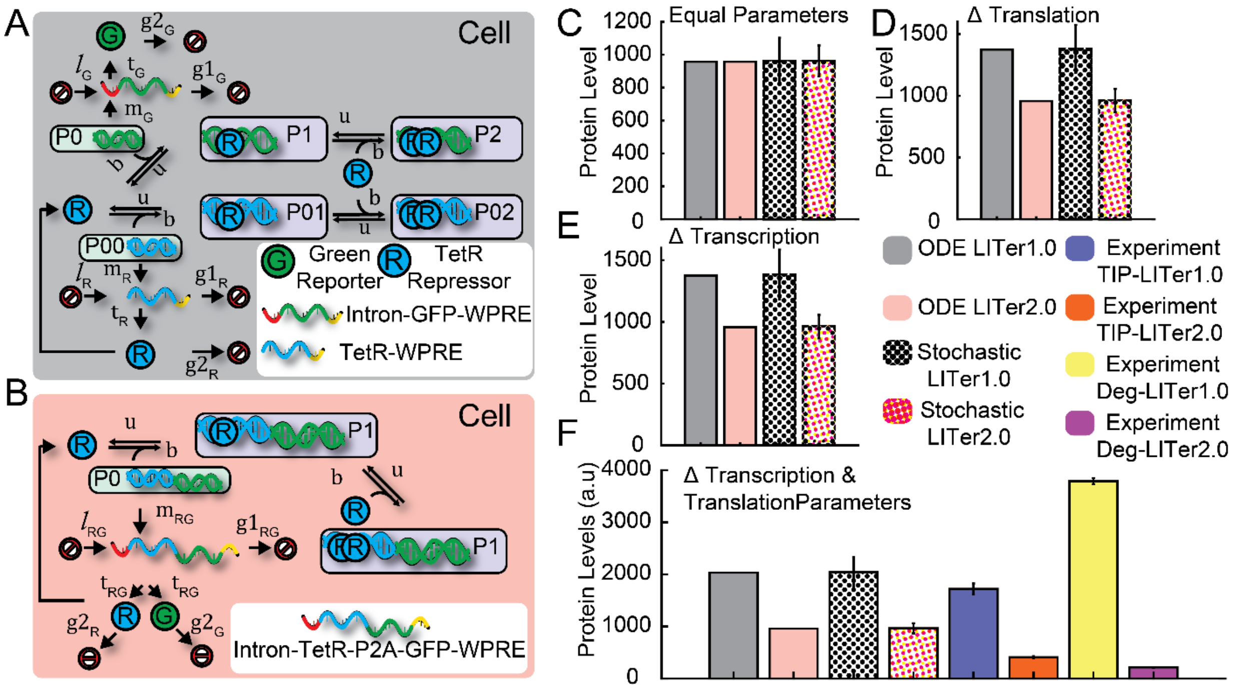

Supporting Figure S8. Deterministic & stochastic models for exploring parameters to lower basal expression. (A) Schematic illustration of relevant gene circuit reactions used for deterministic and stochastic computational model for two promoter LITer system and (B) one promoter LITer system. The deterministic and stochastic model did not distinguish between degradation or TIP mechanism for controlling TetR. See the Supplement for the notation of rates and chemical species. (C) Bar plot representing deterministic and stochastic model predictions for setting equal parameters between the LITer1.0 & LITer2.0 model for basal expression (no induction). (D) Bar plot representing deterministic and stochastic model predictions for changing the translational parameter between the LITer1.0 & LITer2.0 model for basal expression (no induction). (E) Bar plot representing deterministic and stochastic model predictions for changing the transcriptional parameters (synthesis & leakage) between the LITer1.0 & LITer2.0 model for basal expression (no induction). (F) Bar plot representing deterministic and stochastic model predictions for changing both parameters (transcription & translation) between the LITer1.0 & LITer2.0 model for basal expression (no induction) and accompanying experimental data. 10,000 stochastic simulations were run per condition, and experimental results show 3 replicates for each condition. Bars are means and error bars are standard deviation.

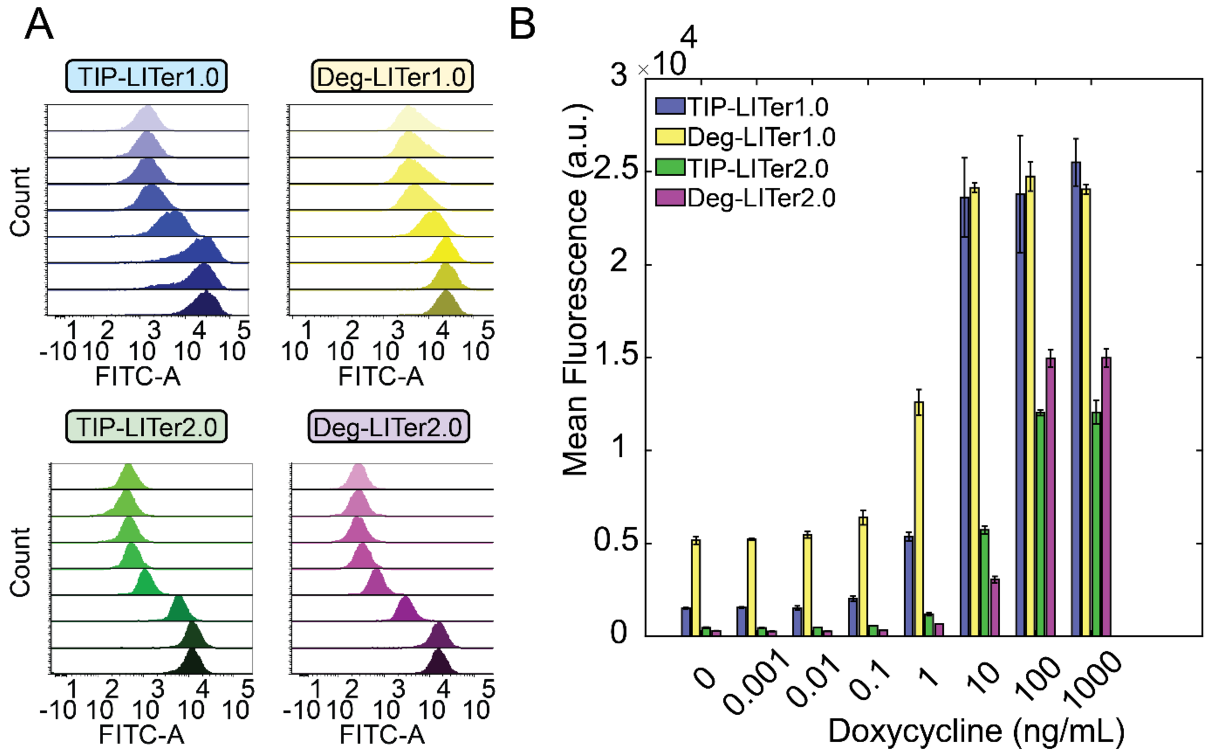

Supporting Figure S9: Gene circuit dose responses to doxycycline induction. (A) Flow cytometry histograms showing chemical induction over a wide range of doses, with fold-induction larger than for light other than the Deg-LITer1.0 which showed similar fold-induction for light. (B) Mean fluorescence intensity of the flow cytometry data, showing up to 51-fold change between no induction and max induction.

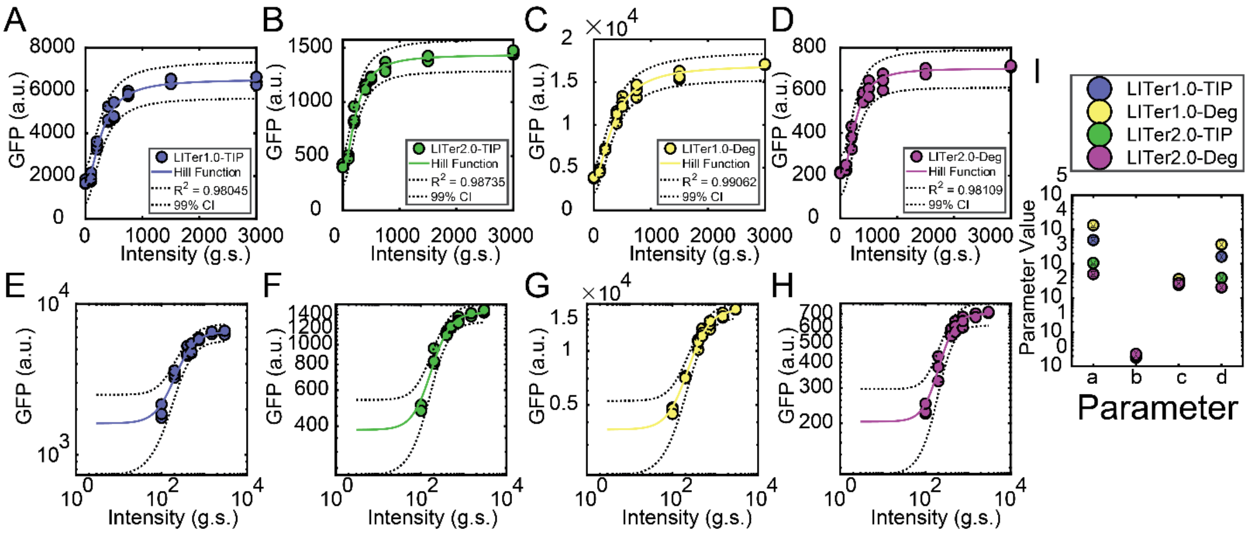

Supporting Figure S10. Hill function fits to mean expression dose-responses. (A-D) Experimental data for light intensity dose response fitted to Hill Function plotted on linear-linear graph. (E-H) Experimental data for light intensity dose response fitted to Hill Function plotted on log-log graph. (I) Parameter values for Hill function fits for LITer systems shown in Supporting Table 4. Circles represent the mean and black arrows represent the confidence intervals.

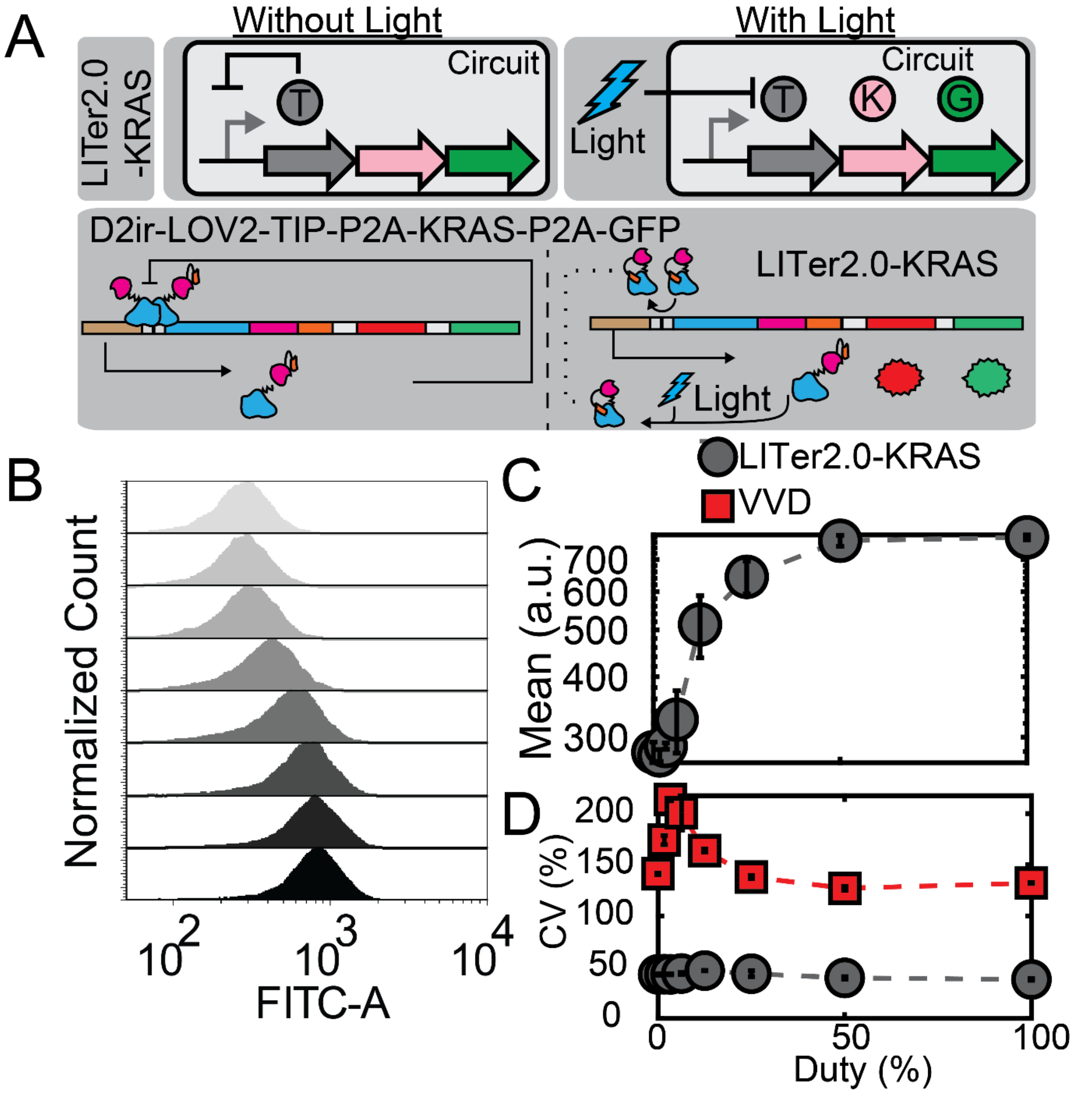

Supporting Figure S11. Duty Cycle of LITer2.0-KRAS. (A) Nodal & Biological schematic illustration of LITer2.0-KRAS circuit under no light & light induction. (B) Flow cytometry histograms of LITer2.0-KRAS gene circuit for duty cycle experiment. (C) Mean fluorescence intensity of LITer2.0-KRAS gene circuit under various duty cycles. (D) CV of LITer2.0-KRAS vs VVD cell line under various duty cycles.

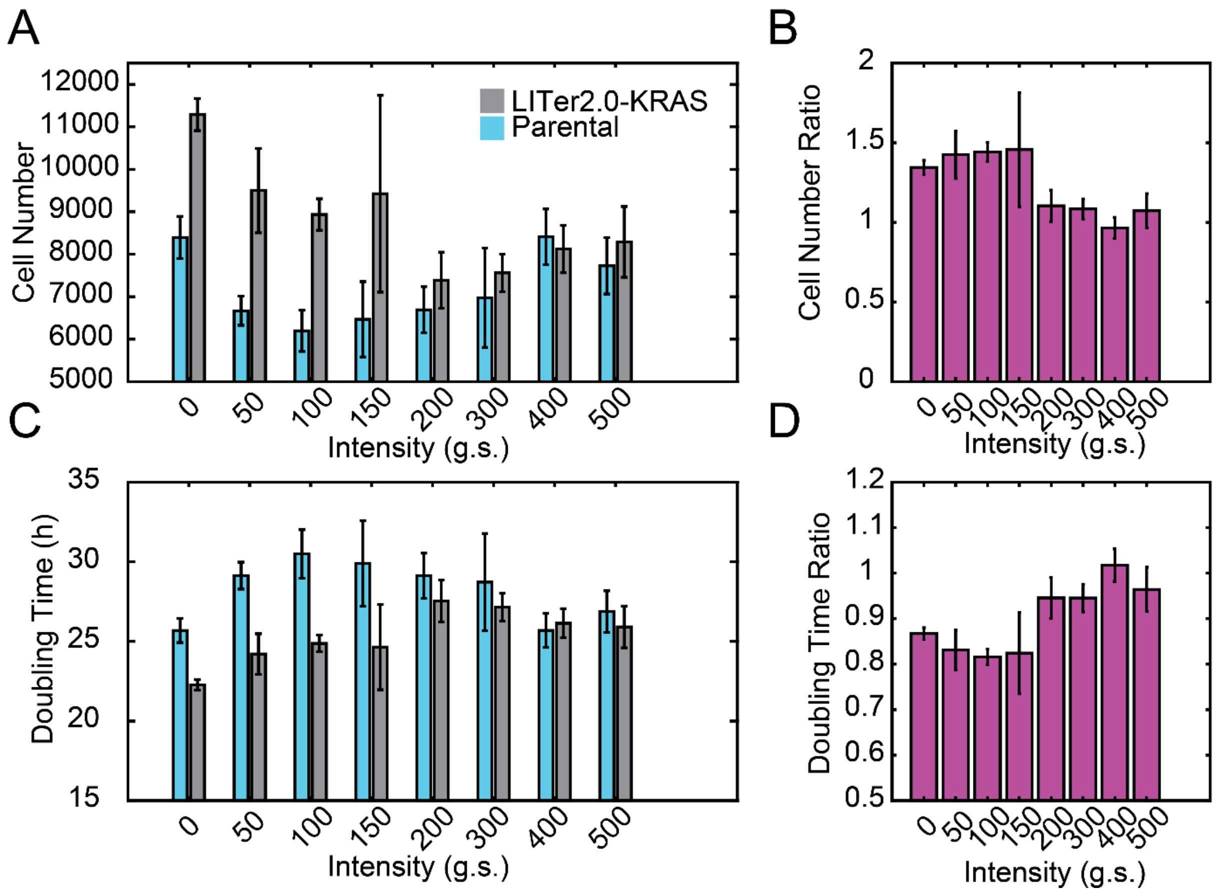

Supporting Figure S12: Growth assay for LITer-KRAS gene circuit.
(A) Cell number count for parental FLP-293 cells & for LITer2.0-KRAS cells under various light induction. (B) Normalized cell number count for LITer2.0-KRAS cells dived by parental cell count under various light induction. (C) Doubling time for parental FLP cells & LITer2.0-KRAS under various light induction. (D) Normalized doubling time expressed as a ratio of LITer2.0-KRAS cell doubling time divided by parental cell doubling time. Cells were seeded at approximately 1200 cells per well and measured via fluorescence microscopy 72h post-induction by NucBlue staining. Three replicates were performed for each condition.

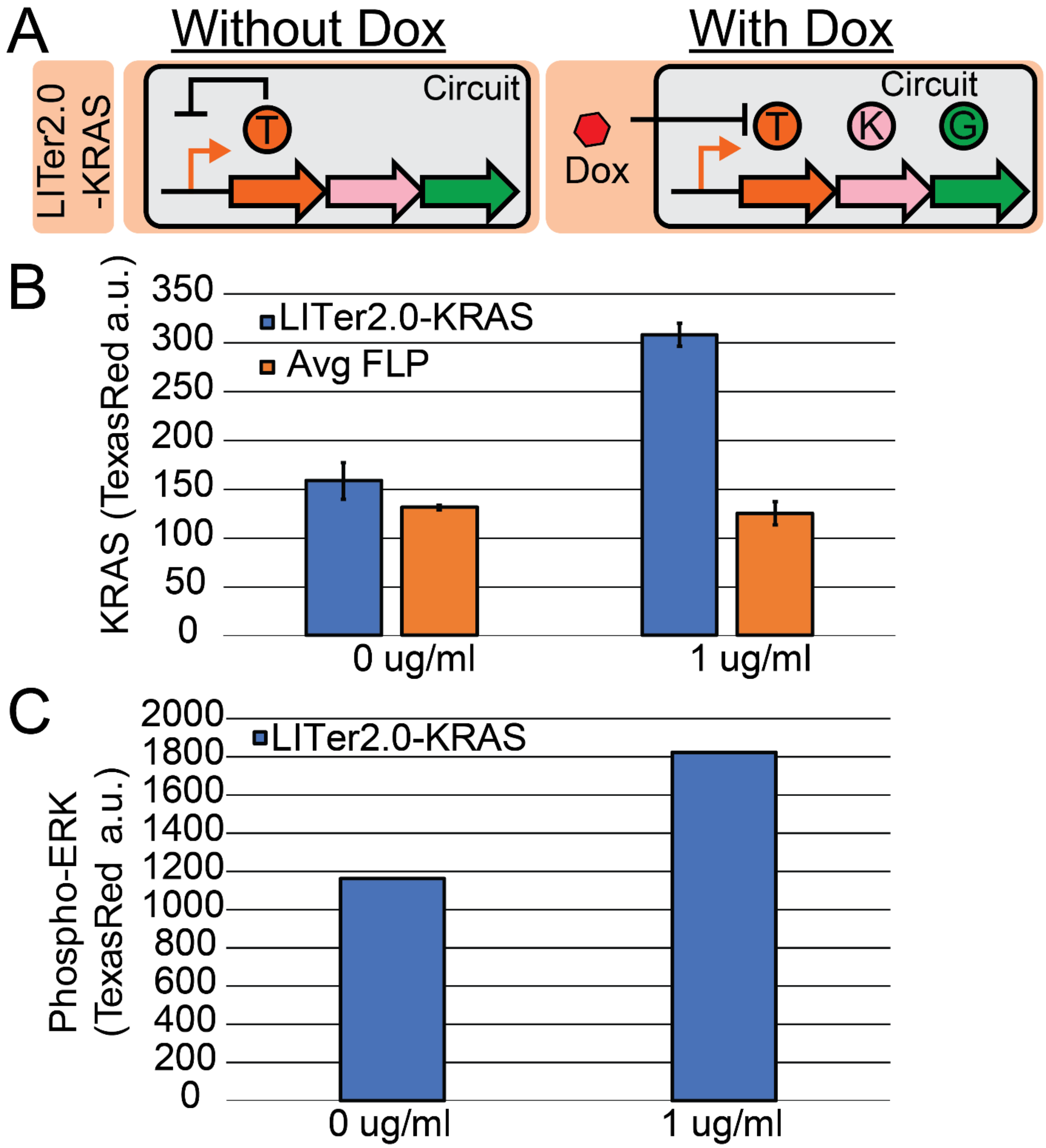

Supporting Figure S13: Doxycycline-induction of KRAS & ERK. (A) Schematic illustration of LITer2.0-KRAS gene circuit. (B) Doxycycline-induction of KRAS in LITer2.0-KRAS cells & parental cell. (C) Doxycycline-induction of ERK in LITer2.0-KRAS cells.

Supporting Tables:

Primer Name	Sequence	Note
P1	CAGTACggatccACCATGGGTTCTAG	BamHI-TetR, forward
P2	gccggcgccgctagcCTTTCTCTTCTTTTTTGGCC	TetR-Lov2 overlap, reverse
P3	GGCCAAAAAAGAAGAGAAAGgctagcggcgccggc	TetR-Lov2 overlap, forward
P4	gtactgACCGGTttaGGAGCCGCCGCCGGAGGGGGCGGCGAAGGCGTAGGCGTTCCAGGTCCAggcggcctcgtcgatg	Lov2-TIP-AgeI, reverse
P5	gtactgACCGGTttagccgcggcggcggg	Lov2-RRRG-degron-AgeI, reverse
P6	caggtcctgcaggcgccaccATGGGTTCTAGACTGGACAAG	hTetR-LOV
P7	tgaagttagtagctccgcttccGGAGCCGCCGCCGGAGG	single promoter TIP
P8	GGTGGCGCCTGCAGGACCTGTAG	pDN-D2irTN2aG5kwh
P9	ggaagcggagctactaacttca	P2A-GFP
P10	TGAAGTTAGTAGCTCCGCTTCCgccgcggcggcgggc	single promoter rrrg
P11	cagtacAAGCTTgccaccATGGTGAGCAAGGGCGAGGA	HindIII-Kozak-tagmNeonGreen, forward
P12	gtactgGGGCCCctaCACCTTCCTCTTCTTCTTAGGCACC	ApaI-NLSx2-tagmNeonGreen, reverse
P13	GTAGCGGCGCATTAAGCGCG	backbone, TG1.55.1
P14	GACGTCAGGTGGCACTTTTC	backbone, TG1.55.1
P15	gaaaagtgccacctgacgtcTCGATAGGTACCGAGTTTCTAGACGG	pU5_fragment
P16	aaggccaggaaccgtaaaaaTACCCCCTAGAGCCCCAGCT	pU5_fragment
P17	agctggggctctagggggtaTTTTTACGGTTCCTGGCCTT	pGAVPO_fragment
P18	cgcgcttaatgcgccgctacTTAAGATACATTGATGAGTTTGGACAAAC	pGAVPO_fragment
P19	GAAAAGTGCCACCTGACGTCcaattgcatgaagaatctgcttag	Positive regulation
P20	cgccggcgccgctagcATAAGATCTGAATTCCCGGGATCCGC	Negative regulation (forward use TG1.17.1)
P21	ctaagcagattcttcatgcaattgGACGTCAGGTGGCACTTTTC	Positive regulation, MV
P22	GCGGATCCCGGGAATTCAGATCTTATgctagcggcgccggcg	Negative regulation, MV (reverse use TG1.17.4)
P23	TGGAGGAGAACCCTGGACCTatgcagattttcgtcaagactttgac	fast-GFP
P24	TGATTGCGGCCGCACCGGctatttgtatagttcatccatgcca	fast-GFP
P25	tggcatggatgaactatacaaatagCCGGTGCGGCCGCAATCA	MV
P26	gtcaaagtcttgacgaaaatctgcatAGGTCCAGGGTTCTCCTCCA	MV
P27	GAAAAGTGCCACCTGACGTCcaattgcatgaagaatctgcttag	Positive regulation
P28	cgccggcgccgctagcCCCGGGGAGCATGTCAAG	Positive regulation
P29	CTTGACATGCTCCCCGGGgctagcggcgccggcg	Positive regulation, MV
P30	ctaagcagattcttcatgcaattgGACGTCAGGTGGCACTTTTC	Positive regulation, MV
P31	TTGACCTTGACATGCTCCCCGGGgctagcggcgccggc	LOV-TIP
P32	GCTGAAGTTAGTAGCTCCGCTTCCggagccgccgccgga	LOV-TIP
P33	CCCGGGGAGCATGTCAAGGTCAA	rtTA, PF
P34	GGAAGCGGAGCTACTAACTTCAGC	P2A
P35	GTTCCAGCTGGGCAGCAGGCGGGCCACGGCCATGATGATCTTGCCGGTggcggcctcgtcgatg	LOV-TCP1, use TG1.16.3 forward, PF
P36	GCCTGCTGCCCAGCTGGAACggaagcggagctactaacttcagc	TCP1-P2A, use TG1.16.1 reverse, PF
P37	CTGCTGCCCAGCTGGAACTAActcgagtctagagggccc	MV, PR
P38	CTCTTGTCCAGTCTAGACATggtgaattcggggccgcgga	MV, PR
P39	tccgcggccccgaattcaccATGTCTAGACTGGACAAGAG	rTetR(rtTA), PR
P40	gggccctctagactcgagTTAGTTCCAGCTGGGCAGCAG	rTetR(rtTA)-LOV-TCP1, PR
P41	TGGAGGAGAACCCTGGACCTatgactgaatataaacttgtggtag	KRAS4B
P42	GCTGAAGTTAGTAGCTCCGCTTCCcataattacacactttgtctttgac	KRAS4B
P43	gtcaaagacaaagtgtgtaattatgGGAAGCGGAGCTACTAACTTCAGC	P2A-GFP (Reverse, use TG1.19.4)
P44	GATTGCGGCCGCACCGGTttacttgtacagctcgtccatgcc	P2A-GFP
P45	ggcatggacgagctgtacaagtaaACCGGTGCGGCCGCAATC	MV
P46	ctaccacaagtttatattcagtcatAGGTCCAGGGTTCTCCTCCA	MV (Mother Vector, TIP, use TG1.19.5)
P47	TGGAGGAGAACCCTGGACCTatgatgcaggacgtgtccagctcg	Twist1
P48	GCTGAAGTTAGTAGCTCCGCTTCCgtgggacgcggacatggacc	Twist1
P49	ggtccatgtccgcgtcccacGGAAGCGGAGCTACTAACTTCAGC	P2A-GFP
P50	cgagctggacacgtcctgcatcatAGGTCCAGGGTTCTCCTCCA	MV

Supporting Table 1. Primers for molecular cloning.

LITer1.0 Stochastic Model Parameters		
**Parameter**	**Role**	**Value**
l_R	**TetR mRNA leakage term**	1
l_G	**GFP mRNA leakage term**	2
m_R	**Maximal TetR mRNA production rate**	100
m_G	**Maximal GFP mRNA production rate**	200
t_R	**Maximal TetR protein production rate**	18.75
t_G	**Maximal GFP protein production rate**	37.5
g_1	**mRNA TetR/GFP degradation/dilution rate**	3.5
g_1	**TetR/GFP Protein degradation/dilution rate**	0.046
b	**TetR binding to TetR/GFP Promoter**	0.2
u	**TetR unbinding to TetR Promoter**	20.8
M_R	**Starting # TetR of mRNA**	0
M_G	**Starting # GFP of mRNA**	0
R	**Starting # of TetR Protein**	0
G	**Starting # of GFP Protein**	0

Supporting Table 2. Model Parameters

LITer2.0 Stochastic Model Parameters		
Parameter	Role	Value
$\boldsymbol{l_{RG}}$	mRNA leakage term	$\mathbf{2}$
$\boldsymbol{m_{RG}}$	Maximal mRNA production rate	$\mathbf{200}$
$\boldsymbol{t_{RG}}$	Maximal protein production rate	$\mathbf{37.5}$
$\boldsymbol{g_1}$	mRNA degradation/dilution rate	$\mathbf{3.5}$
$\boldsymbol{g_2}$	Protein degradation/dilution rate	$\mathbf{0.046}$
$\boldsymbol{b}$	TetR binding to TetR/GFP Promoter	$\mathbf{0.2}$
$\boldsymbol{u}$	TetR unbinding to TetR/GFP Promoter	$\mathbf{20.8}$
$\boldsymbol{M_{RG}}$	Starting # of mRNA	$\mathbf{0}$
$\boldsymbol{R}$ & $\boldsymbol{G}$	Starting # of TetR or GFP Protein	$\mathbf{0}$

Supporting Table 3.

Parameters	Mean	99% CI Min	99% CI Max
a	4911.38	4443.17	5379.58
b	1.89	1.46	2.32
c	295.11	252.55	337.67
d	1615.28	1305.66	1924.89
a	13345.17	12409.35	14281.00
b	1.81	1.50	2.11
c	355.24	319.86	390.62
d	3649.48	3088.80	4210.15
a	1051.24	974.88	1127.61
b	1.97	1.62	2.33
c	240.50	212.86	268.14
d	383.99	329.51	438.47
a	499.05	454.76	543.35
b	2.30	1.77	2.83
c	269.12	234.38	303.86
d	203.88	172.21	235.56

Supporting Table 4. Hill Function fit parameters for TIP-LITer1.0 (blue), Deg-LITer1.0 (yellow), TIP-LITer2.0 (green), Deg-LITer2.0 (magenta).

Supporting References:

BIBLIOGRAPHY AND REFERENCES CITED

1 Nevozhay, D., Zal, T. & Balazsi, G. Transferring a synthetic gene circuit from yeast to mammalian cells. *Nat Commun* **4**, 1451, doi:10.1038/ncomms2471 (2013).

2 Muller, K., Zurbriggen, M. D. & Weber, W. An optogenetic upgrade for the Tet-OFF system. *Biotechnol Bioeng* **112**, 1483-1487, doi:10.1002/bit.25562 (2015).

3 Wang, X., Chen, X. & Yang, Y. Spatiotemporal control of gene expression by a light-switchable transgene system. *Nat Methods* **9**, 266-269, doi:10.1038/nmeth.1892 (2012).

4 Shaner, N. C. *et al.* A bright monomeric green fluorescent protein derived from Branchiostoma lanceolatum. *Nat Methods* **10**, 407-409, doi:10.1038/nmeth.2413 (2013).

5 Ahearn, I. M., Haigis, K., Bar-Sagi, D. & Philips, M. R. Regulating the regulator: post-translational modification of RAS. *Nat Rev Mol Cell Biol* **13**, 39-51, doi:10.1038/nrm3255 (2011).

6 Nevozhay, D., Adams, R. M., Murphy, K. F., Josic, K. & Balazsi, G. Negative autoregulation linearizes the dose-response and suppresses the heterogeneity of gene expression. *Proc Natl Acad Sci U S A* **106**, 5123-5128, doi:10.1073/pnas.0809901106 (2009).

7 Gerhardt, K. P. *et al.* An open-hardware platform for optogenetics and photobiology. *Sci Rep* **6**, 35363, doi:10.1038/srep35363 (2016).
